# Impact of SARS-CoV-2 Outbreak on Emergency Department Presentation and Prognosis of Patients with Acute Myocardial Infarction: A Systematic Review and Updated Meta-Analysis

**DOI:** 10.3390/jcm11092323

**Published:** 2022-04-21

**Authors:** Emma Altobelli, Paolo Matteo Angeletti, Francesca Marzi, Fabrizio D’Ascenzo, Reimondo Petrocelli, Giuseppe Patti

**Affiliations:** 1 Department of Life, Public Health and Environmental Sciences, University of L’Aquila, 67100 L’Aquila, Italy; paolomatteoangeletti@gmail.com (P.M.A.); francesca.marzi@univaq.it (F.M.); 2 Cardiac Surgical Intensive Care Unit, Giuseppe Mazzini Hospital, 64100 Teramo, Italy; 3 Cardiovascular and Thoracic Department, Division of Cardiology, University of Turin, 10126 Turin, Italy; fabrizio.dascenzo@unito.it; 4 San Timoteo Hospital, ASREM Molise, 86039 Termoli, Italy; r.petrocelli@libero.it; 5 Department of Translational Medicine, Maggiore della Carità Hospital, University of Eastern Piedmont, 28100 Novara, Italy; giuseppe.patti@uniupo.it

**Keywords:** SARS-CoV-2, STEMI, NSTEMI, meta-analysis, acute myocardial infarction, geographical areas

## Abstract

We performed an updated meta-analysis to robustly quantify admission trends of patients with ST-segment elevation MI (STEMI) and non-ST-segment elevation MI (NSTEMI) during the first wave of the pandemic and to characterize on a large basis the risk profile and early prognosis. Studies having the same observation period for the comparison between SARS-CoV-2 outbreak in 2020 versus control period in 2019 were included. Primary endpoints were the relative variation of hospital admissions, the difference of in-hospital mortality for STEMI and NSTEMI. Secondary were: mortality according to countries, income levels and data quality; cardiogenic shock, mechanical complications, door-to-balloon time, time from symptom onset to first medical contact, left ventricular ejection fraction (LVEF) and troponin. In total, 61 observational studies with 125,346 patients were included. Compared with 2019, during the pandemic for STEMI were observed: a 24% reduction of hospitalizations with an impact on early survival (OR = 1.33 in-hospital mortality); the time from symptom onset to first medical contact was 91.31 min longer, whereas door-to-balloon time was increased (+5.44 min); after STEMI, the rate of cardiogenic shock was 33% higher; LVEF at discharge was decreased (−3.46); elevated high-sensitivity troponin levels (1.52) on admission. For NSTEMI, in the COVID-19 period, we observed a 31% reduction of hospitalizations and higher in-hospital deaths (OR = 1.34). The highest mortality rates among countries were: Italy OR = 3.71 (high income), Serbia OR = 2.15 (upper middle) and Pakistan OR = 1.69 (lower middle). Later hospital presentation was associated with larger infarctions, as well as with increased cardiogenic shock and in-hospital mortality.

## 1. Introduction

In December 2019, the outbreak of the CoronaVirus Disease-19 (COVID-19), an acute respiratory distress syndrome caused by a new coronavirus (SARS-CoV-2), started in China [1]. The viral diffusion then spread, with a pandemic state declared by the World Health Organization on 11 March 2020 [2,3]. COVID-19 is still widely diffused, representing a health concern across a majority of countries in the world. During this pandemic state, various studies have shown a significant reduction of Emergency Department (ED) presentations for acute cardiac diseases requiring in-hospital management, such as acute coronary syndromes (ACS) [4,5,6,7,8,9], severe rhythm disturbances needing pace-maker implantation [10] or congestive heart failure [11]. Citizens’ underestimation of cardiac symptoms along with the fear of contagion in the EDs could explain this pattern. In the setting of ACS, hospital admissions generally decreased for both ST-segment elevation myocardial infarction (STEMI) and non-STEMI (NSTEMI). This reduction was prevalent when a national lockdown with social-containing measures was approved [12]. Indeed, other large-sized observational investigations did not demonstrate any reduction in admissions for STEMI during the SARS-CoV-2 pandemic [13,14]. Therefore, based on the results of individual studies, it may be difficult to accurately characterize COVID-19-related patterns of ED presentations for MI, especially across different geographic regions and different pandemic periods. Moreover, it has been observed that patients admitted for ACS, especially for STEMI, during the SARS-CoV-2 outbreak had a poorer prognosis and higher early mortality compared to those in the corresponding period in 2019 [9,15]. To date, no pooled data on in-hospital deaths in patients admitted for NSTEMI have been published. Finally, single studies may be underpowered to assess outcomes related to adverse events at low incidence (e.g., post-infarction complications).

Pooled analyses of data from multiple investigations can provide more robust data on the abovementioned clinically relevant issues. We performed an updated analysis of observational studies to quantify on a large basis the impact of the SARS-CoV-2 outbreak on the rates and features of patients admitted to the ED for MI and on the early outcome of such patients.

## 2. Methods

We used the following strict inclusion criteria to reduce the chance of bias: (1) studies involving patients presenting STEMI and NSTEMI, and (2) the same duration of the observation period for the comparison between the COVID-19 period in 2020 and the control period, defined “pre-pandemic period”, before the first COVID-19 case was diagnosed in each country.

Ethics Committee approval was not required because our study was based on a meta-analysis of aggregated published data.

The literature research was conducted on PubMed, EMBASE, Scopus, Science Direct, Web of Science and Cochrane database registry on 6 January 2022. The keywords for each database are reported in Table S1. Manual research was also conducted. Editorials and reviews from major medical journals published within the last two years were also searched for further information on studies of interest. Only investigations published in the English language were considered.

The papers were selected by two independent reviewers (P.M.A. and G.P.); a methodologist (E.A.) resolved any disagreements. The selection of the studies was made using the PRISMA 2020 guidelines (Figure S1) [16]. Quality control of the systematic review was performed using the PRISMA checklist (Table S2). For case-control and cohort studies, the bias analysis was performed using the Newcastle–Ottawa Scale [17] (Table S3). References of included studies are reported in Table S4 and references excluded with reasons are showed in Table S5.

Meta-regression analyses were utilized for the following variables: % of females, mean age, % of diabetic patients, % of current smokers, % of hypertense patients, % of dyslipidemias among patients, % of patients with chronic kidney disease and countries. Meta-regressions were performed if the numbers of studies reporting the variables of interest were ≥4.

### Study Endpoints

The primary endpoint was the relative variation of hospital admissions for STEMI and NSTEMI during the COVID-19 period in 2020 compared to corresponding time frames in 2019. The co-primary endpoint was in-hospital mortality in patients admitted for STEMI and NSTEMI during the COVID-19 period in 2020 vs. corresponding time frames in 2019.

Secondary outcome measures were the following variations during the COVID-19 period in 2020 versus corresponding time frames in 2019:

- Hospital admissions for STEMI and NSTEMI according to different countries, pandemic periods, income levels according to World Bank [18]: high income (HI), upper–middle income (UMI) and lower–middle income (LMI), and data quality on three levels, according to methodology reported by Globocan [19].
- Time from symptom onset to first medical contact, door-to-balloon time, left ventricular ejection fraction (LVEF) on admission and at discharge, troponin values at admission and at discharge in STEMI patients.
- In-hospital cardiogenic shock and mechanical complications in STEMI patients.

When information about an outcome of interest was unavailable, the study was not used for such an endpoint.

Regarding meta-analysis, Odds Ratio (OR) and Incidence Rate Ratio (IRR) were used for dichotomic outcomes; standardized mean difference (MD) with 95% CI and related p-value was used as a measure of effect size for continuous data. Cohen’s d was used to describe the standardized mean difference of troponin effect. Cohen’s value can be used to compare effects across studies, even when the dependent variables are measured in different ways [20].

Data from primary studies presented as medians and interquartile ranges were transformed into mean and standard deviation (SD), as described by Pudar Hozo et al. [21]. A random effects model was used to account for different sources of variation among studies. Heterogeneity was assessed using Q statistics and I^2^. Publication bias was analyzed and represented by a funnel plot, with funnel plot symmetry assessed with Egger’s test. Publication bias was checked using the trim and fill procedure. STATA 16 version software was used.

## 3. Results

Research of the electronic databases according to the above-listed criteria found 61 papers, whereas the manual search found none. The records removed before screening were: 18,997 duplicate records, 2407 records marked as ineligible by automation tools and 626 records removed for other reasons. Of the 113 investigations assessed for eligibility, 52 reports were excluded for the following reasons: 27 did not report the outcome of interest, 6 were previous reviews or meta-analyses and 19 considered different calendar periods (Figure S1). A total of 14 studies came from Italy, 8 from China, 5 from Germany, 4 from Israel and Turkey, 3 from France, 2 from the Helvetic Confederation, India, Poland, Spain, US and UK, 1 from Albania, Austria, Egypt, Greece, Iran, Ireland, Japan, Pakistan, Portugal, Saudi Arabia and Canada.

There were 51,680 patients included in the studies during the pandemic period and 73,666 for the control period. The list of included studies is reported in Table S3, while the studies excluded with reasons were reported in Table S4.

**Figure 1:**
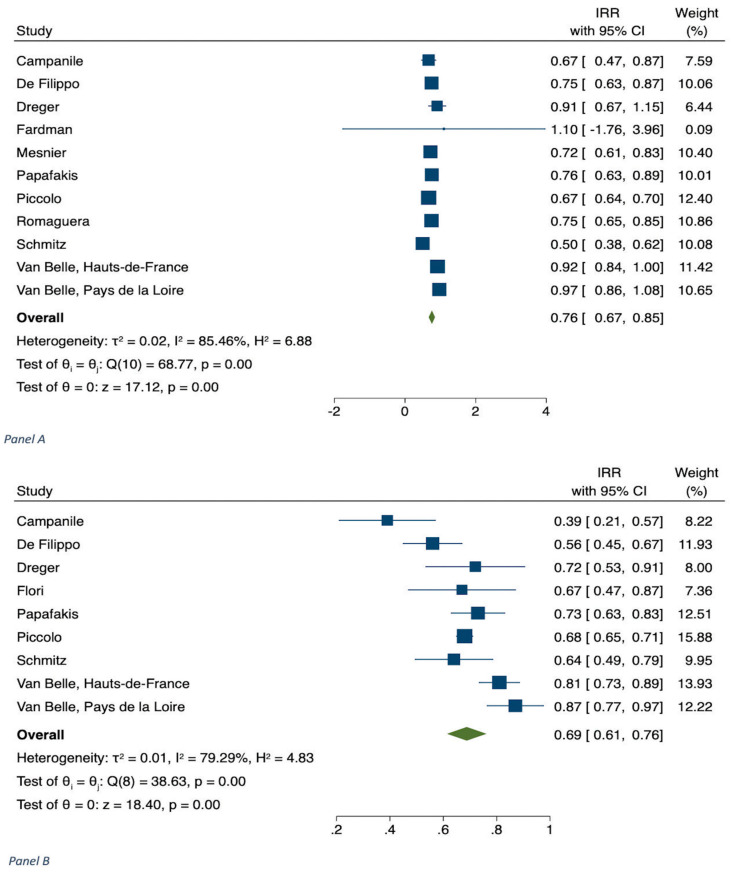
Panel (**A**) STEMI patients: Hospital admissions during COVID-19 pandemic in 2020 vs. corresponding control period. Panel (**B**) NSTEMI patients: Hospital admissions during COVID-19 pandemic in 2020 vs. corresponding control period.

**Figure 2:**
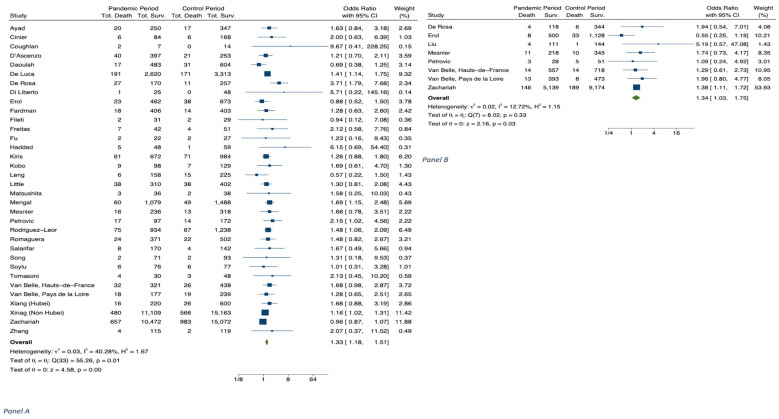
Panel (**A**) STEMI patients: Hospital mortality during COVID-19 pandemic in 2020 vs. corresponding control period. Panel (**B**) NSTEMI patients: Hospital mortality during COVID-19 pandemic in 2020 vs. corresponding control period.

**Figure 3:**
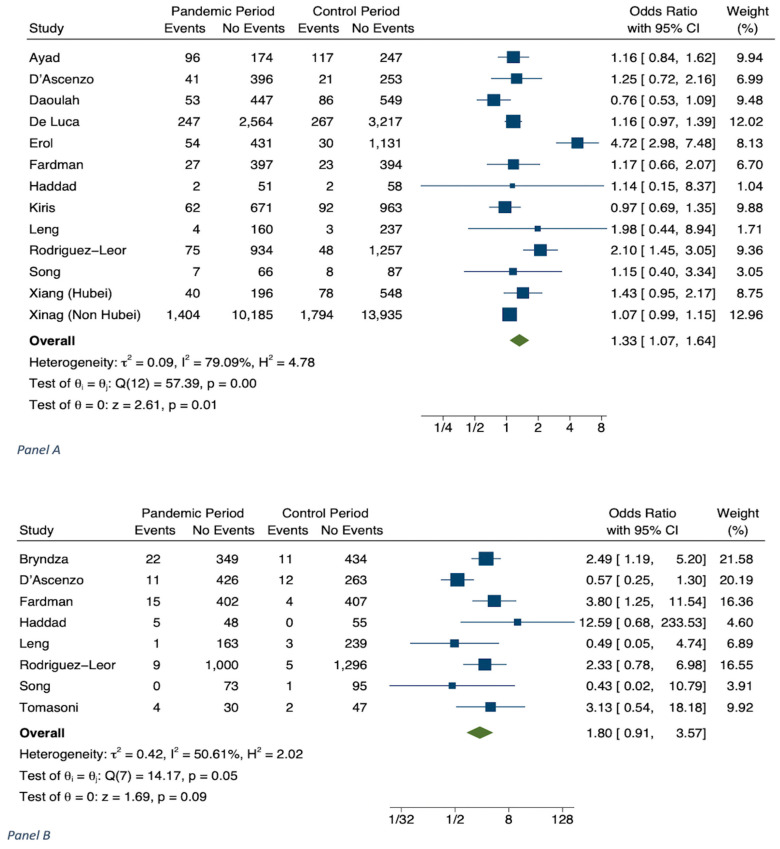
STEMI patients. Panel (**A**): Occurrence of cardiogenic shock during COVID-19 pandemic in 2020 vs. corresponding control period. Panel (**B**): Occurrence of mechanical complications during COVID-19 pandemic in 2020 vs. corresponding control period.

**Figure 4:**
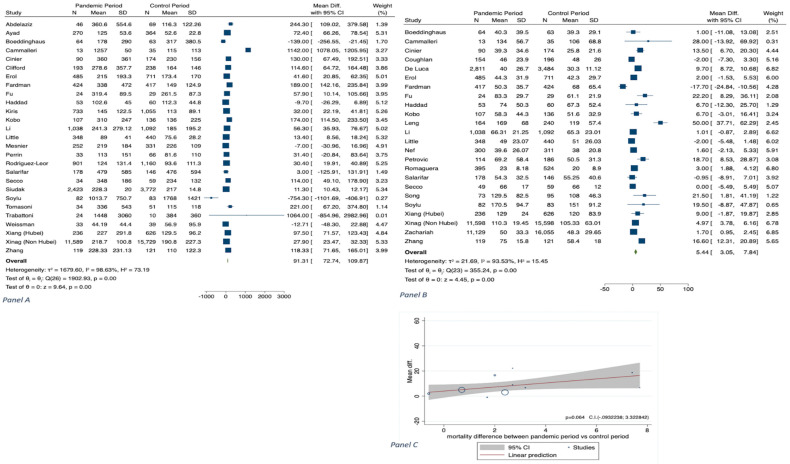
Panel (**A**): MD in time (minutes) from symptom onset to first medical contact. Panel (**B**): MD in door-to-balloon time (minutes). Panel (**C**): Meta-regression analysis: relationship between differences in door-to-balloon time and mortality during COVID-19 pandemic in 2020 vs. corresponding control period.

**Figure 5:**
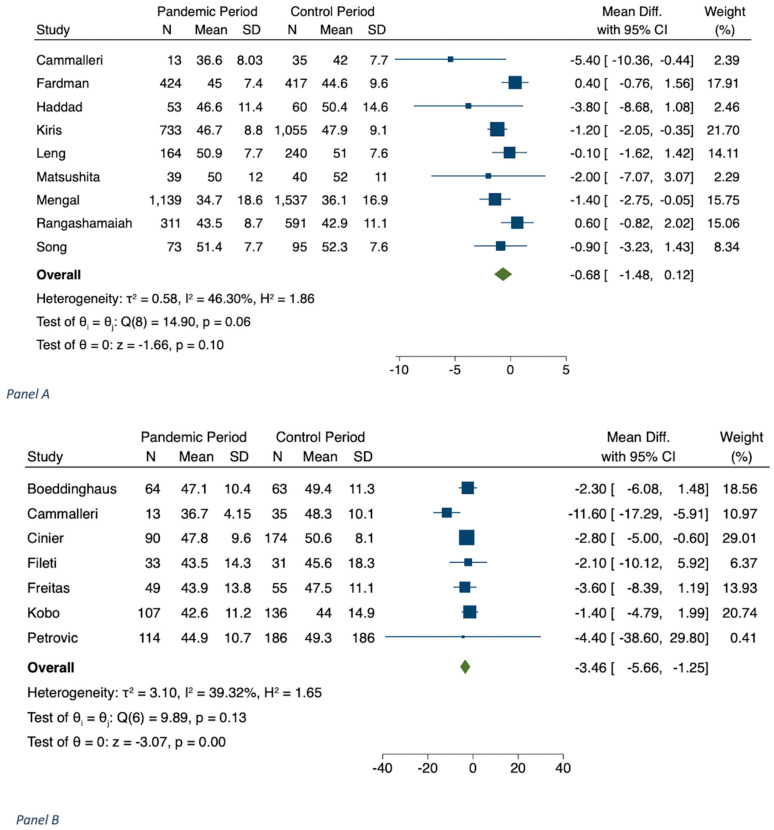
Panel (**A**): MD of LVEF (%) on hospital admission. Panel (**B**): LVEF (%) at discharge during COVID-19 pandemic in 2020 vs. corresponding control period.

The results of meta-analyses are shown in Table 1, in Figure 1, Figure 2, Figure 3, Figure 4 and Figure 5 and in Figure S2.

Hospital admissions of STEMI and NSTEMI patients during the COVID-19 pandemic versus the corresponding control period are reported in Table S5.

Meta-regression results are reported in Table S6. Meta-regressions according to countries for hospital admission and mortality are shown in Figures S4 and S5, respectively. Subgroup analyses according to income levels and data quality are indicated in Figures S6 and S7, respectively.

### 3.1. Primary and Secondary Outcomes in STEMI

The number of hospitalizations, expressed as IRR, for STEMI during the COVID-19 outbreak in 2020 versus the control period was estimated from 11 studies (n = 10,082) (Table 1) [4,14,22,23,24,25,26,27,28,29]. This comparison showed a decrease during the pandemic, with an IRR of 0.76 (CI 0.67–0.85, *p* < 0.001; I^2^ = 85.46) (Table 1 and Figure 1). As regards hospital admissions, we also evaluated from 3 studies (n = 859) the difference between earlier versus later phases of the pandemic [12,22,29]. Our results demonstrated no statistical significance (Figure S2).

Mortality for STEMI during the COVID-19 period in 2020 versus the control period was estimated from 34 studies (n = 79,682) [7,14,15,24,25,28,30,31,32,33,34,35,36,37,38,39,40,41,42,43,44,45,46,47,48,49,50,51,52,53,54,55]. We found a significantly higher in-hospital mortality in 2020, with OR 1.33 (CI. 1.18–1.51, *p* < 0.001; I^2^ = 40.28) (Table 1 and Figure 2). Concerning gender, meta-regression showed no significant increase in mortality (Figure S3).

The analysis on cardiogenic shock in hospitalized STEMI patients during the COVID-19 period in 2020 versus 2019 was performed from 13 studies (n = 44,229) [15,24,30,33,34,36,40,41,43,48,50,53] We observed an increase in the former period, with OR 1.33 (CI 1.07–1.64, *p* = 0.001) (Table 1 and Figure 3A). The OR for STEMI-related mechanical complications in 2020 versus 2019, performed on 8 studies (N = 5461), was 1.80 (CI 0.91–3.57, *p* = 0.09) (Table 1 and Figure 3B) [15,24,40,43,48,50,52,56]. A total of 28 studies considered the time from symptom onset to first medical contact (minutes) (n = 46384). In the COVID-19 period, there was a significant delay of 91.31 min (CI 72.74–109.87, *p* < 0.001; I^2^ = 98.63) (Table 1 and Figure 4A) [24,25,30,31,36,39,40,41,42,44,48,49,51,52,53,55,56,57,58,59,60,61,62,63,64,65,66]. We found a relationship between the time of first medical contact and smoking and CKD, respectively (k = 18, *p* = 0.015; k = 4, *p* = 0.029) (Table S6).

There were 24 studies that reported door-to-balloon time (n = 70,914). Door-to-balloon time was longer during the pandemic than before (MD+5.44, CI 3.05–7.84, *p* < 0.001; I^2^ = 93.53) (Table 1 and Figure 4B) [13,24,31,32,34,36,39,40,42,43,44,47,48,49,50,51,53,54,55,60,61,62,63]. However, there was no significant difference in mortality during 2020 versus the control period observed in relation to differences in door-to-balloon time (Figure 4C).

A total of 9 studies (n = 7019) reported LVEF at admission [21,24,40,41,43,45,46,50,61,67] and 7 (n = 1150) at discharge [32,38,39,43,48,62,63]. LVEF at admission was not associated with a significant percentage decrease of this parameter during the COVID-19 period versus the preceding year: −0.66 (−1.49; 0.16, *p* = 0.116); on the other hand, LVEF at discharge was associated with a significant percentage decrease in 2020 versus 2019: −3.46 (−5.66; −1.25, *p* < 0.001) (Table 1 and Figure 5).

Troponin at baseline was significantly higher in the COVID-19 period versus 2019, especially for high-sensitivity troponin (Cohen’s = 1.52, CI 1.03–2.24, *p* < 0.001) (Table 1) [31,37,38,42,45,59,60,61]. As regards the troponin peak, we did not find a statistically significant difference (Table 1) [42,50,52,62,69].

Moreover, we analyzed geographical differences among countries. We performed a meta-regression on hospital admissions according to country (Figure S4) and a meta-regression on mortality according to country and income levels (Figures S5 and S6, respectively).

As regards hospital admissions, we showed no significant differences by country; indeed, they decreased in most countries (Q = 6.84, *p* = 0.23), with Italy (IRR = 0.68, CI 0.64–0.71) and Germany (IRR = 0.69, CI 0.29–1.09) showing the lowest hospital admissions (Figure S4).

On the other hand, meta-regression analysis showed a significant increase in mortality rates among countries analyzed (*p* = 0.003). The highest mortality rate was in Serbia (OR = 2.15), followed by Italy (OR = 1.97), Pakistan and France, with OR values of 1.69 and 1.55, respectively (Figure S5).

Subgroup analysis for mortality according to income level (HI, LMI and UMI) showed no statistically significant differences among countries (*p* = 0.08) (Figure S6).

However, among the HI countries, the highest mortality rate was in Italy (OR = 3.71, CI 1.79–7.68), the highest among the UMI was in Serbia (OR = 2.15, CI 1.02–4.56) and the highest among LMI was in Pakistan (OR = 1.69, CI 1.15–2.48) (Figure S5). It is important to note that Italy and Serbia present a high data quality (Figure S7).

### 3.2. Primary and Secondary Outcomes in NSTEMI

The hospitalization for NSTEMI during the COVID-19 period in 2020 versus the corresponding control period was estimated from 11 studies (n = 10,182) (Table 1) [4,14,22,23,26,27,29,68]. There was a significant decrease of hospital admissions (Figure 1B), with an IRR 0.69 (CI 0.61–0.76), *p* < 0.001; I^2^ = 79.29). In addition, we evaluated mortality from 8 studies (N = 19,910); this analysis showed a significant increase in mortality, with an OR of 1.34 (1.03–1.75, *p* = 0.002), without heterogeneity (I^2^ = 12.72, *p* = 0.33) (Table 1, Figure 2B) [7,14,25,36,47,54,69].

Concerning hospital admissions in NSTEMI patients, 3 studies (n = 766) reported the difference between earlier versus later phases of the pandemic; they demonstrated no statistical significance (Figure S2) [12,22,29].

Regarding hospital admissions, our results showed that Italy had the lowest value for NSTEMI with IRR = 0.59 and CI = 0.47–0.71 (Figure S8). Finally, meta-regression showed no significant difference for mortality among the countries analyzed (*p* = 0.19) (Figure S9).

Finally, the references reported in Table S5 [70,71,72,73,74] were included in a systematic review, but not in a meta-analysis because they did not present the outcomes of interest.

## 4. Discussion

To the best of our knowledge, this meta-analysis on 61 studies and 125,346 patients is the largest report from an epidemiologic and prognostic point of view that explores the impact of the COVID-19 pandemic on patients suffering from MI, with a specific differentiation between STEMI and NSTEMI. The most relevant results for STEMI are: (1) hospitalizations were significantly reduced compared to the corresponding periods in the preceding year, with a detrimental impact on early survival; (2) the time from symptom onset to first medical contact was longer, while door-to-balloon time did not markedly change; (3) this later presentation was associated with an increased risk of mechanical complications, mainly cardiogenic shock, and was associated with a lower ejection fraction, as well as more elevated levels of high-sensitivity troponin at admission. Admissions for NSTEMI were similarly reduced, with an increased risk of death.

Acute respiratory infections have been historically regarded as a trigger for acute cardiovascular events [75]. This was recently confirmed by a meta-analysis by Caldeira et al., reporting a fivefold higher MI risk in patients suffering from severe influenza compared to the general population [76]. Altogether, this evidence is strongly discordant with the results of the present meta-analysis, where, when compared with the corresponding periods in the preceding year, a remarkable reduction in hospitalizations for MI was observed during the COVID-19 outbreak. This was consistent across different countries. A biological explanation for such a phenomenon is unlikely: viral-induced up-regulation of inflammatory cytokines and systemic inflammation in patients with SARS infection would be expected to provide a pro-coagulant state, increasing the likelihood of coronary plaque rupture and acute coronary syndrome. As a matter of fact, previous coronaviruses outbreaks were associated with a significant burden of cardiovascular morbidities and complications [77]. Similarly, COVID-19 is characterized by a systemic inflammatory response that leads to endothelial dysfunction and oxidative stress, with consequent hemostatic activation, in terms of both platelet hyper-reactivity and the triggering of coagulation cascade [78]. The clinical translation of these changes is represented by a risk >3 times higher of multivessel coronary thrombosis demonstrated in COVID-19 patients with STEMI and by a more relevant thrombotic burden at the site of the culprit coronary vessel [79]. Thus, the reduction of hospitalizations for MI should be related to citizens’ behavioral response to the pandemic scenario. Specifically, in our meta-analysis, we observed a 24% decrease in admissions for STEMI and 31% for NSTEMI during COVID-19 outbreak versus the corresponding time frames in 2019.

Alarmingly, epidemiological data suggests that approximately one-fourth to one-third of MI patients, in very large areas of the globe, during the COVID-19 pandemic in 2020, remained at home and did not have access to PS. Consistently, observational data from Northern Italy indicated an increase in the number of cases of out-of-hospital cardiac arrest following the temporal trend of the COVID-19 epidemic outbreak [80]. In light of the results of our meta-analysis, without going to hospital. Furthermore, our findings may have epidemiological relevance as they may hypothesize an increase in the number of people with post-acute cardiological problems in the coming period, related to the development of dilated ischemic cardiomyopathy in survivors of myocardial infarction at home, without reperfusion, during the COVID-19 outbreak. The decrease in hospitalizations appears mainly due to the fear of contagion in the emergency rooms, despite the fact that hospitals have designed specific procedures aimed at protecting against interindividual viral transmission, and the underestimation of cardiac symptoms.

The present meta-analysis raises two other major points of interest, which may assist both physicians and the healthcare system. First, a continuing impact of COVID-19 on hospital admissions over time. In fact, the decrease of hospitalizations, especially for STEMI, versus 2019 was observed during both earlier phases of the first COVID-19 wave. Second, our data indicate that during the pandemic in 2020, the prolongation of door-to-balloon time was limited, e.g., 5.4 min (upper limit of CI = 7.8 min) compared with the preceding year. Notably, a meta-regression found no relationship between variation in door-to-balloon time and the difference in mortality between 2020 and 2019. This appears of particular interest: despite the logistic and technical difficulties related to SARS-CoV-2 protection issues for patients and operators with dedicated procedures and devices, the time from hospital admission to cath lab was not markedly increased, stressing the preserved performance of the STEMI pathways, despite the overall critical situation. This surely does not support other strategies alternative to primary percutaneous coronary intervention in STEMI patients, such as thrombolysis, which have recently been proposed [54].

Fear of contagion and the underestimation of symptoms during the COVID-19 pandemic also led to a significant delay of ED presentation in STEMI patients compared with the 2019 control periods. In particular, the time from symptom onset to first medical contact was 91 min longer. According to previous evidence indicating that the beneficial effect of reperfusion in STEMI is higher in patients arriving within 2 h after symptom onset versus those arriving later [81], the extent of delay observed in our work precluded, in most cases, patients from drawing the maximal benefit from recanalization of the culprit vessel. In fact, STEMI patients had larger infarctions at ED presentation, as demonstrated by increased high-sensitivity troponin levels on admission and impaired ejection fraction at discharge (−2.3%), with consequent worse short- and long-term prognoses. In fact, in-hospital mortality among STEMI patients in 2020 versus 2019 was 33% higher. Notably, when compared with the preceding year, the survival rate was also lower after NSTEMI (−34%) during the COVID-19 period.

Mechanical complications and cardiogenic shock in STEMI are a function of the delay in the vessel recanalization. Such events can dramatically impact the prognosis of patients suffering from non-promptly treated MI. It is estimated that 10% to 15% of in-hospital deaths in STEMI patients are attributable to mechanical complications (mainly cardiac rupture and tamponade), with cardiogenic shock being responsible for most of the remainder [82]. The late admission to ED for patients reporting chest pain represents the most relevant predictor of cardiogenic shock in the case of STEMI; accordingly, we demonstrated during the COVID-19 outbreak a significant increase of cardiogenic shock and an increase of mechanical complications (the latter did not reach statistical significance). Another worrisome issue is that, based on the present analysis, a higher proportion of patients discharged after STEMI during the COVID-19 period with a low ejection fraction may develop congestive heart failure during follow-up due to ischemic dilated cardiomyopathy.

With reference to STEMI, geographical differences among countries in relationship to hospital admission did not reveal statistically significant differences. Hospital mortality subgroup analysis considering income levels (HI, UMI and LMI) showed no statistically significant differences, because it increased in most of them. However, among the HI countries, the highest mortality rates were in Italy and Spain, while the highest among the UMI and LMI countries was in Serbia and Pakistan, respectively.

The present paper has some limitations. As regards a comparison among countries, it is necessary to make some considerations. In fact, data from geographic areas relating to some countries included in the meta-analysis, both for hospital admissions and mortality rates, should be treated with due caution for reasons also related to the quality of data collection. If we consider the quality of the mortality data, not all the countries analyzed can be classified as equally accurate. Some death rates may therefore be underestimated, such as in Egypt, India, Pakistan or Saudi Arabia. The latter, which is high-income, is considered not to have a high-quality data collection method, so it could be assumed that the data are underestimated. Importantly, continually improving the quality of epidemiological data collection provides crucial support to public health decision makers.

Second, the prolongation of the time to admission was detectable, but the prolongation of medical contact was not specifically quantifiable in all cases, potentially impacting the results. Third, the meta-analysis includes only papers written in English.

In conclusion, a meta-analysis on a similar topic has recently been published [9]. However, it did not focus on variations of admissions for ACS during the COVID-19 period and across different outbreak waves, it did not consider NSTEMI patients and did not investigate the risk profile of STEMI patients at admission in relation to troponin levels; it did not perform a correlation between the variation of door-to-balloon time and the difference of mortality in 2020 versus 2019; and it included only half of the studies comprised in our meta-analysis. Notably, the time from symptom onset to first medical contact was 91 min in our work compared with 38 min in Chew’s paper.

Finally, our paper may represent a robust snapshot, containing a quantification of COVID-19-related missing hospitalizations for MI, the risk profile of patients admitted for STEMI during the pandemic in 2020, the impact of their later presentation on prognosis and the effectiveness of dedicated in-hospital pathways for the urgent treatment of patients with acute coronary syndromes. Moreover, this evidence might help healthcare systems manage and assist an expected higher number of people coming to the hospitals for severe, post-acute cardiological issues in the future.

## Figures and Tables

**Table 1:** Meta-analysis results: pandemic period 2020 versus control period.

STEMI								
**Outcomes**	**K**	**Total Sample Size**	**IRR** ***p***	**I^2^** ***p***	**Egger’s** ***p***	**Begg and Mazumdar’s** ***p***	**Fail Safe** ***n***	**Rosenthal’s** ***n***
Number of hospital admissions [4,14,22,23,24,25,26,27,28,29].	11	10082	0.76 (0.67; 0.85) *p* < 0.001	85.46 *p* < 0.001	0.654	0.876	435	65
			**OR** ***p***	**I^2^** ***p***	**Egger’s** ***p***	**Begg and Mazumdar’s** ***p***	**Fail Safe** ***n***	**Rosenthal’s** ***n***
Overall mortality [7,14,15,24,25,28,30,31,32,33,34,35,36,37,38,39,40,41,42,43,44,45,46,47,48,49,50,51,52,53,54,55]	34	79682	1.33 (1.18; 1.51) *p* < 0.001	40.28 *p* = 0.01	0.057	0.150	580	180
Cardiogenic shock [15,24,30,33,34,36,40,41,43,48,50,53]	13	44229	1.33 (1.07; 1.64) *p* = 0.01	79.09 *p* < 0.001	0.288	0.807	20	75
Mechanical complications [15,24,40,43,48,50,52,56].	8	5461	1.80 (0.91; 3.57) *p* = 0.09	50.61 *p* = 0.05	0.727	0.805	12	50
Length of stay [24,33,37,42,43,44,45,47,51,52,57,58]	12	4678	1.02 (0.72; 1.43) *p* = 0.922	98.80 *p* < 0.001	0.752	0.681	0	70
	**K**	**Total Sample Size**	**MD**	**I^2^** ***p***	**Egger’s** ***p***	**Begg and Mazumdar’s** ***p***	**Fail Safe** ***n***	**Rosenthal’s** ***n***
Time to first medical contact (min) [24,25,30,31,36,39,40,41,42,44,48,49,51,52,53,55,56,57,58,59,60,61,62,63,64,65,66].	28	46384	91.31 (72.74; 109.87) *p* < 0.001	98.63 *p* < 0.001	0.042	0.323	6208	150
Door-to-balloon time (min) [13,24,31,32,34,36,39,40,42,43,44,47,48,49,50,51,53,54,55,60,61,62,63].	24	70914	5.44 (3.05; 7.84) *p* < 0.001	93.53 *p* < 0.001	0.286	0.102	1443	130
LVEF on admission (%) (*) [21,24,40,41,43,45,46,50,61,67]	9	7019	−0.66 (−1.49; 0.16) *p* = 0.06	46.30 *p* = 0.100	0.226	0.084	1573	135
LVEF at discharge (%) [32,38,39,43,48,62,63]	7	1150	−3.46 (−5.66; −1.25) *p* < 0.001	39.32 *p* = 0.130	0.775	0.453	56	45
	**K**	**Total Sample Size**	**Cohen’s d**	**I^2^** ***p***	**Egger’s** ***p***	**Begg and Mazumdar’s** ***p***	**Fail Safe** ***n***	**Rosenthal’s** ***n***
Troponin at baseline * [30,33,42,45,59,60,61]	9	3400	0.40 (0.26; 0.55) *p* = 0.026	66.95 *p* = 0.002	0.068	0.404	19	55
High-sensitivity Troponin * [42,50,52,59,60,61]	6	1388	1.52 (1.03; 2.24) *p* < 0.001	6.04 *p* = 0.380	-	-	-	-
Other/not specified Troponin * [30,32,42]	3	2012	0.32 (−0.36; 0.09) *p* = 0.520	44.68 *p* = 0.00	-	-	-	-
Troponin peak * [42,44,50,59,62]	5	666	−0.11 (−0.33; 0.12) *p* = 0.354	43.85 *p* = 0.07	0.695	1.0	0	35
**NSTEMI**								
**Outcomes**	**K**	**Total Sample Size**	**IRR** **Incidence Rate Ratio**	**I^2^** ***p***	**Egger’s** ***p***	**Begg and Mazumdar’s** ***p***	**Fail Safe** ***n***	**Rosenthal’s** ***n***
Number of hospital admissions [4,14,22,23,26,27,29,68].	11	10182	0.69 (0.61; 0.76) *p* < 0.001	79.29 *p* < 0.001	0.007	0.036	147	65
	**K**	**Total Sample Size**	**OR** ***p***	**I^2^** ***p***	**Egger’s** ***p***	**Begg and Mazumdar’s** ***p***	**Fail Safe** ***n***	**Rosenthal’s** ***n***
Overall mortality [7,14,25,37,48,55,69].	8	19910	1.34 (1.03; 1.75) *p* = 0.03	12.72 *p* = 0.33	0.521	0.521	11	50

COVID-19 = CoronaVirus Disease-19; K = number of primary studies; I^2^ = I-square; IRR = Incidence Rate Ratio; LVEF = left ventricular ejection fraction; OR = Odds Ratio; MD = mean difference; STEMI = ST-segment elevation myocardial infarction, NSTEMI = non-ST-segment elevation myocardial infarction, (* ng/dL.)

## Data Availability

Data are included in the primary studies.

## Supplementary Materials

The following supporting information can be downloaded at: https://www.mdpi.com/article/10.3390/jcm11092323/s1. Figure S1. Selection of studies by PRISMA 2020 guidelines. Figure S2. STEMI and NSTEMI patients. Hospital admissions difference between earlier and later phases of the pandemic. Figure S3. STEMI. Meta-regression analysis according to gender. Figure S4. STEMI. Meta-regression analysis (ORs, 95% CI): hospital admissions according to country. Figure S5. STEMI. Meta-regression analysis (ORs, 95% CI): mortality according to country. Figure S6. STEMI. Mortality subgroup analysis (ORs, 95% CI) according to income levels. Figure S7. STEMI. Mortality subgroup analysis (ORs, 95% CI) according to data quality. Figure S8. NSTEMI. Hospital admission subgroup analysis (ORs, 95% CI) according to country. Figure S9. NSTEMI. Mortality subgroup analysis (ORs, 95% CI) according to country. Table S1. Research strategy. Table S2. PRISMA checklist. Table S3. Included studies, calendar weeks, quality assessment and outcomes. Table S4. References of reports excluded with reasons. Table S5. Hospital admissions of STEMI and NSTEMI patients during COVID-19 pandemic in 2020 vs. corresponding control period. Table S6. Meta-regression results.

## Abbreviations

COVID-19	CoronaVirus Disease-19
IRR	Incidence Rate Ratio
LVEF	Left ventricular ejection fraction
ACS	Acute coronary syndrome
ED	Emergency department
OR	Odds ratio
MD	Mean difference
CI	Confidence interval
SD	Standard deviation
STEMI	ST-segment elevation myocardial infarction
NSTEMI	Non-ST-segment elevation myocardial infarction
MI	Myocardial infarction
HI	High income
UMI	Upper–middle income
LMI	Lower–middle income
