# Supplementary material for: Impact of SARS-CoV-2 Outbreak on Emergency Department Presentation and Prognosis of Patients with Acute Myocardial Infarction: A Systematic Review and Updated Meta-Analysis
Source: J Clin Med. 2022 Apr 21;11(9):2323. doi: 10.3390/jcm11092323 (PMC9102296; doi:10.3390/jcm11092323)
Supplement: Supplementary file 1 [file jcm-11-02323-s001.zip › jcm-1653479-SI.pdf]

Supplementary Materials

Supplementary Figures

Figure S1. Selection of studies by PRISMA 2020 guidelines.
Figure S2. STEMI and NSTEMI patients. Hospital admissions difference between earlier and later phases of the pandemic.
Figure S3. STEMI. Meta-regression analysis according to gender.
Figure S4. STEMI. Meta-regression analysis (ORs, 95% CI): hospital admissions according to country.
Figure S5. STEMI. Meta-regression analysis (ORs, 95% CI): mortality according to country.
Figure S6. STEMI. Mortality subgroup analysis (ORs, 95% CI) according to income levels.
Figure S7. STEMI. Mortality subgroup analysis (ORs, 95% CI) according to data quality.
Figure S8. NSTEMI. Hospital admission subgroup analysis (ORs, 95% CI) according to country.
Figure S9. NSTEMI. Mortality subgroup analysis (ORs, 95% CI) according to country.

Supplementary Tables

Table S1. Research strategy.
Table S2. PRISMA checklist.
Table S3. Included studies, calendar weeks, quality assessment and outcomes.
Table S4. References of reports excluded with reasons.
Table S5. Hospital admissions of STEMI and NSTEMI patients during COVID-19 pandemic in 2020 vs. corresponding control period.
Table S6. Meta-regression results.

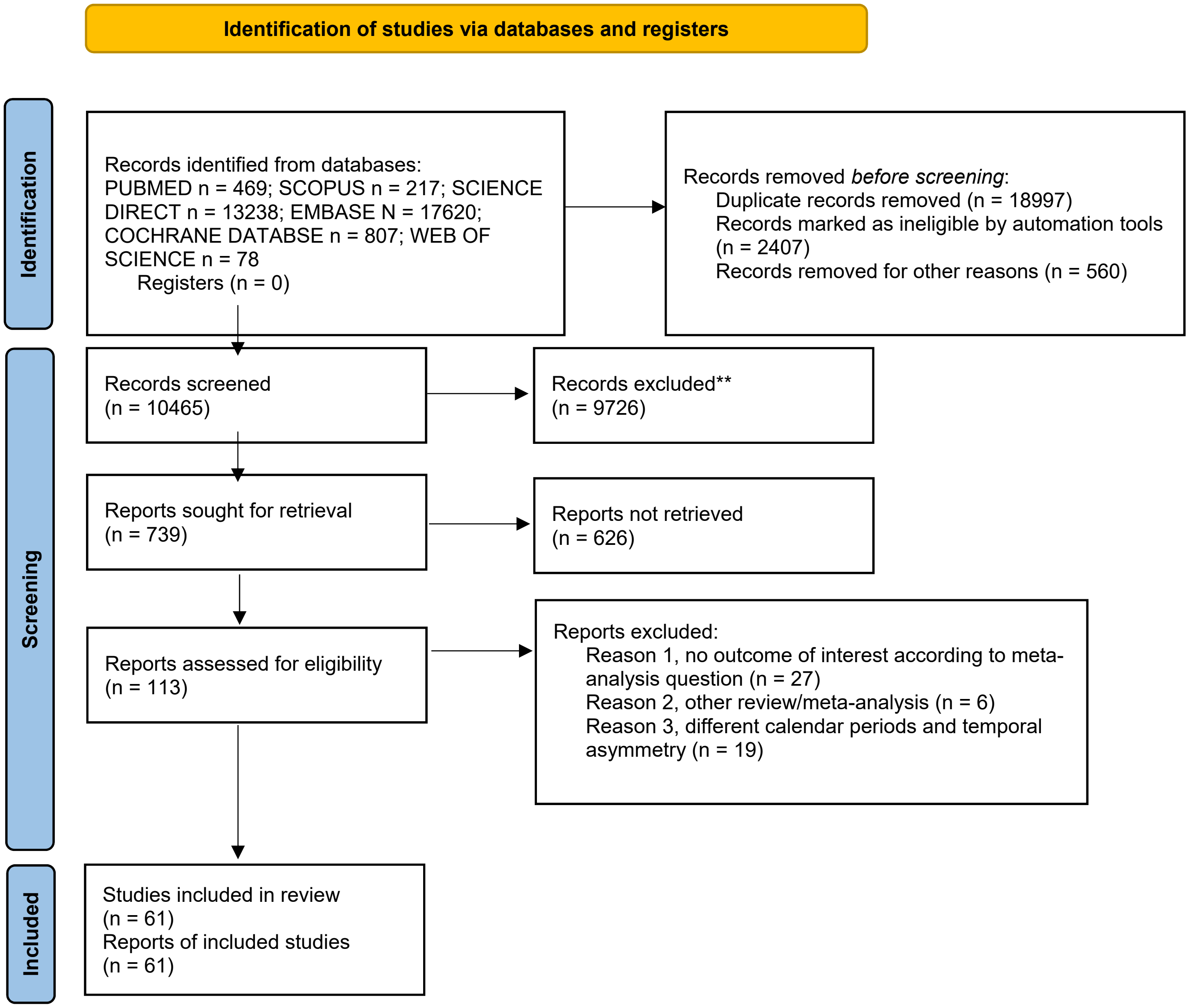

Figure S1. Selection of studies by PRISMA 2020 guidelines.

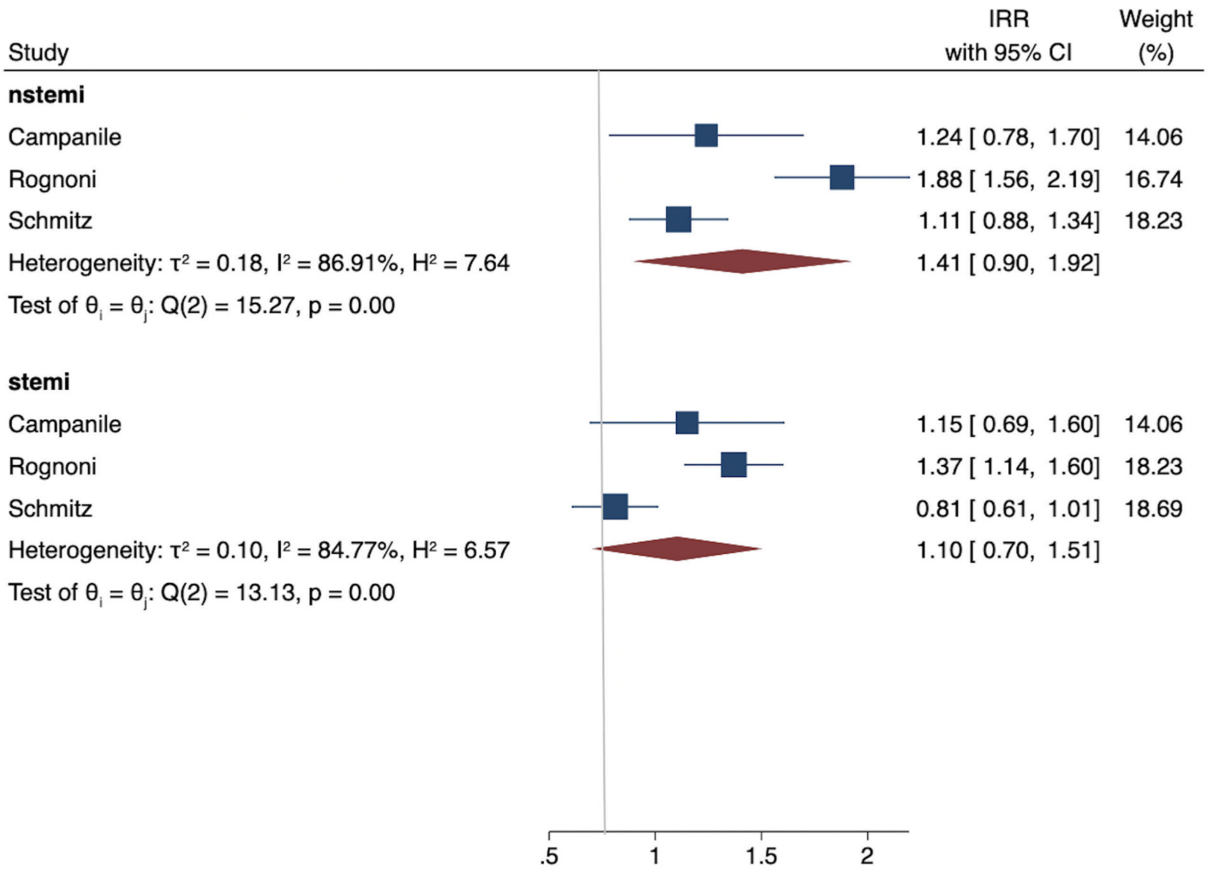

Figure S2. STEMI and NSTEMI patients. Hospital admissions difference between earlier and later phases of the pandemic.

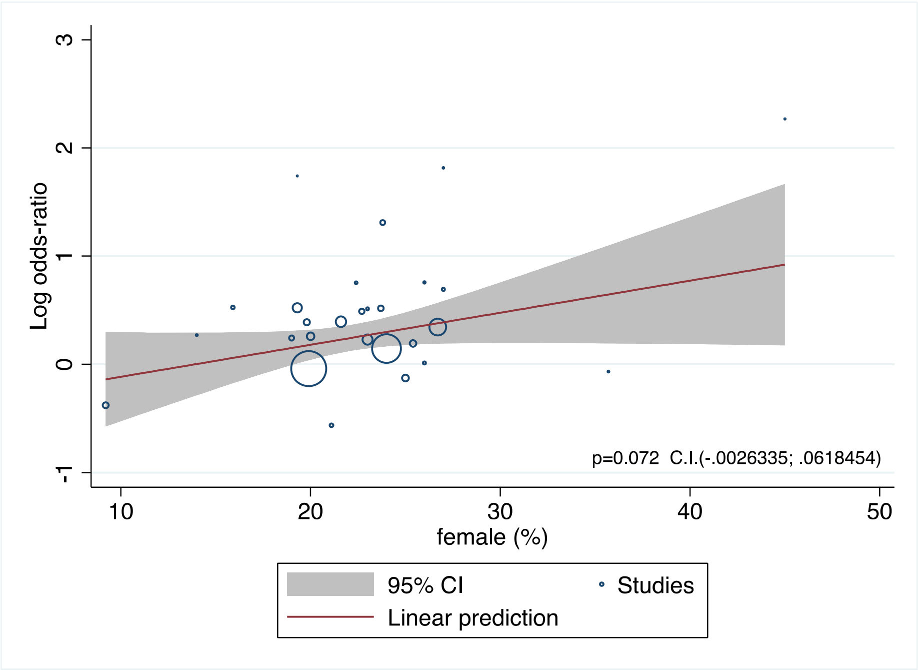

Panel A

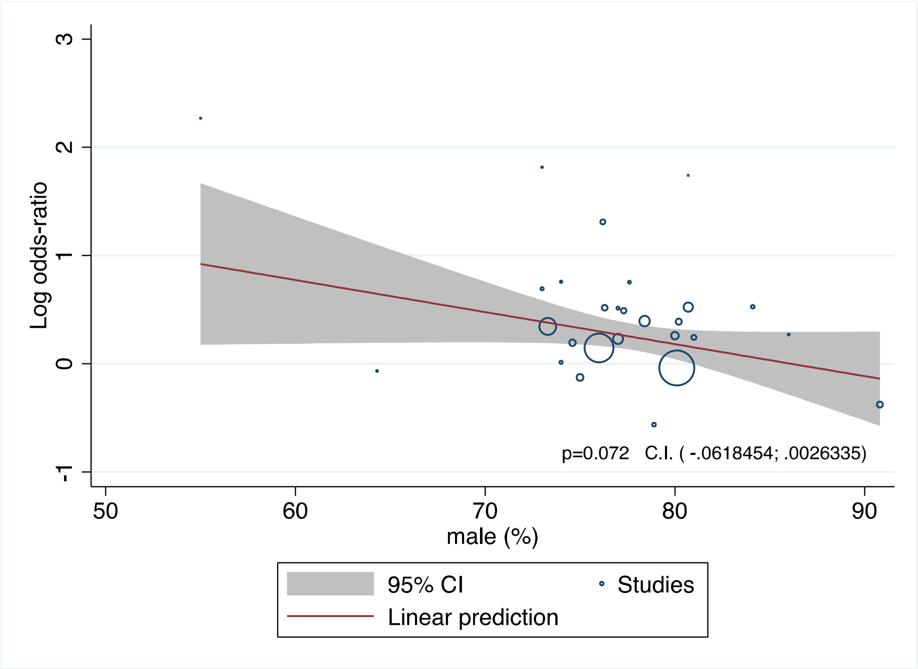

Panel B

Figure S3. STEMI. Meta-regression analysis according to gender. Panel **(A)** Female Gender; Panel **(B)** Male Gender.

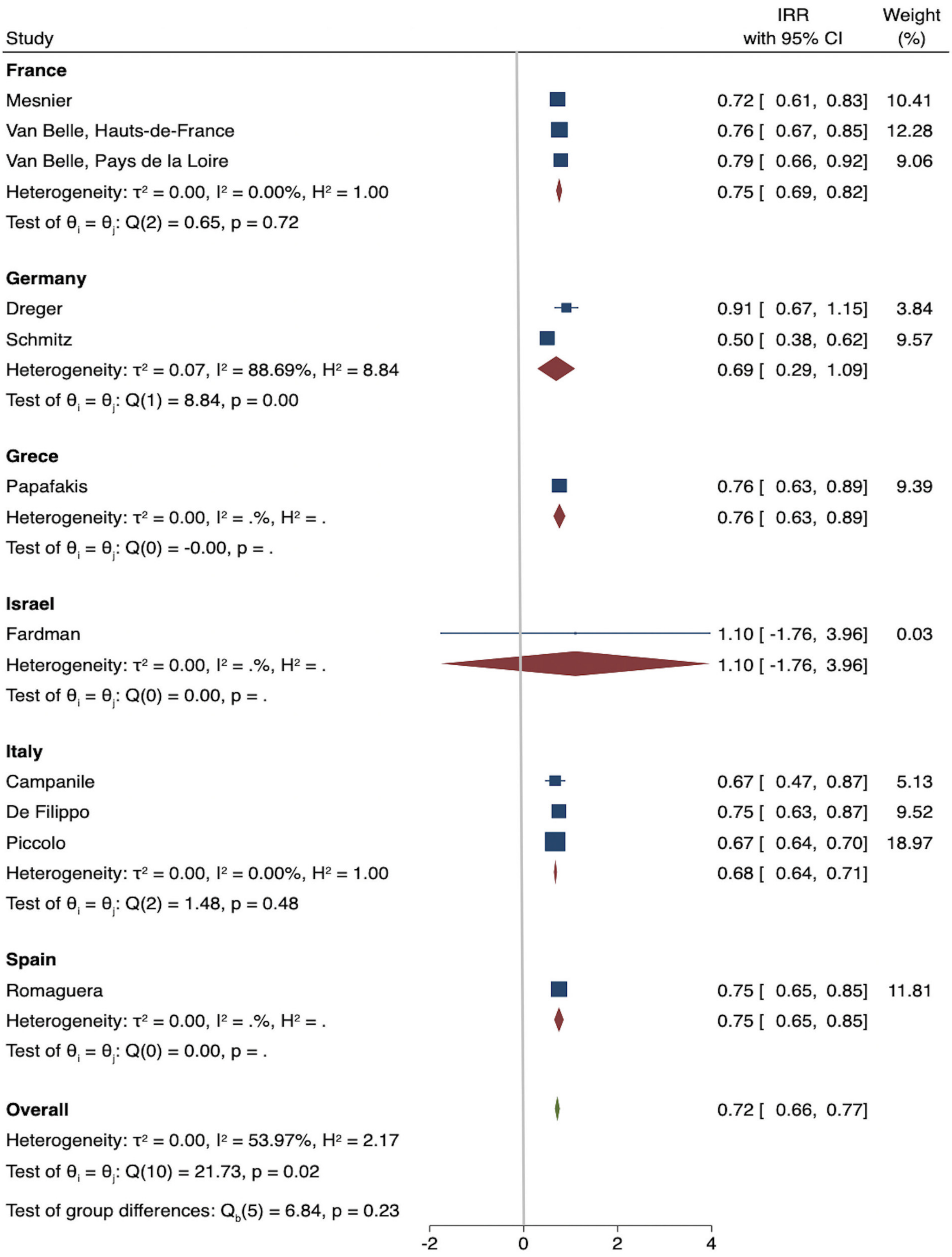

Figure S4. STEMI. Meta-regression analysis (ORs, 95% CI): hospital admissions according to country.

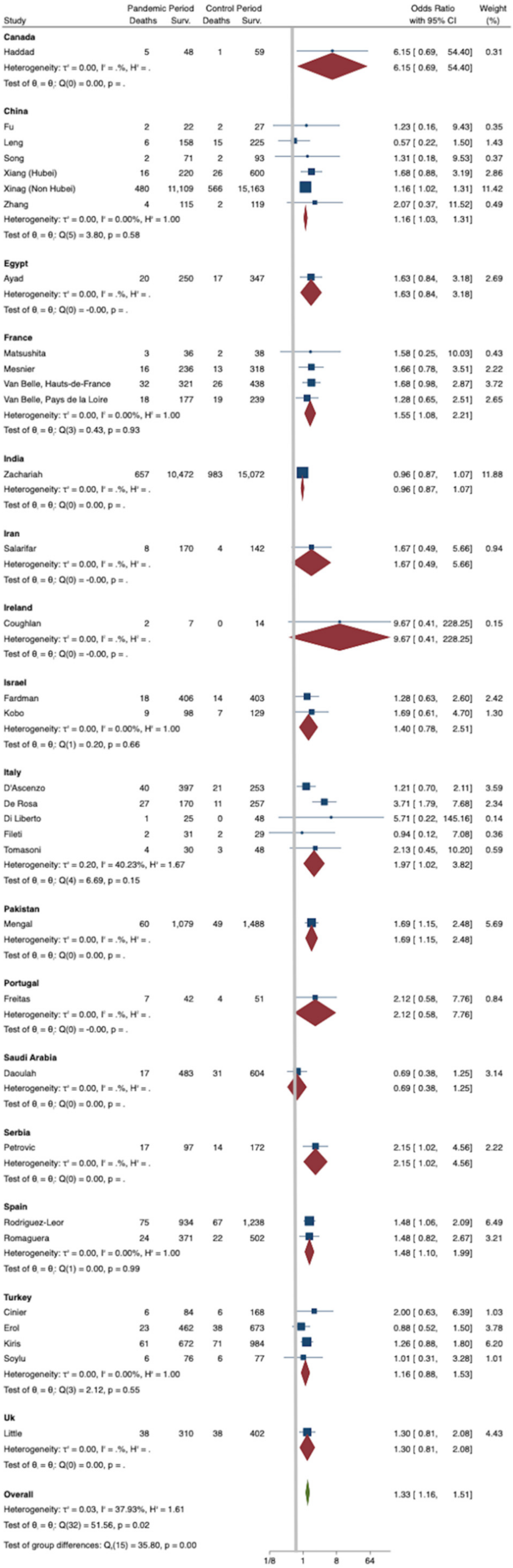

Figure S5. STEMI. Meta-regression analysis (ORs, 95% CI): mortality according to country.

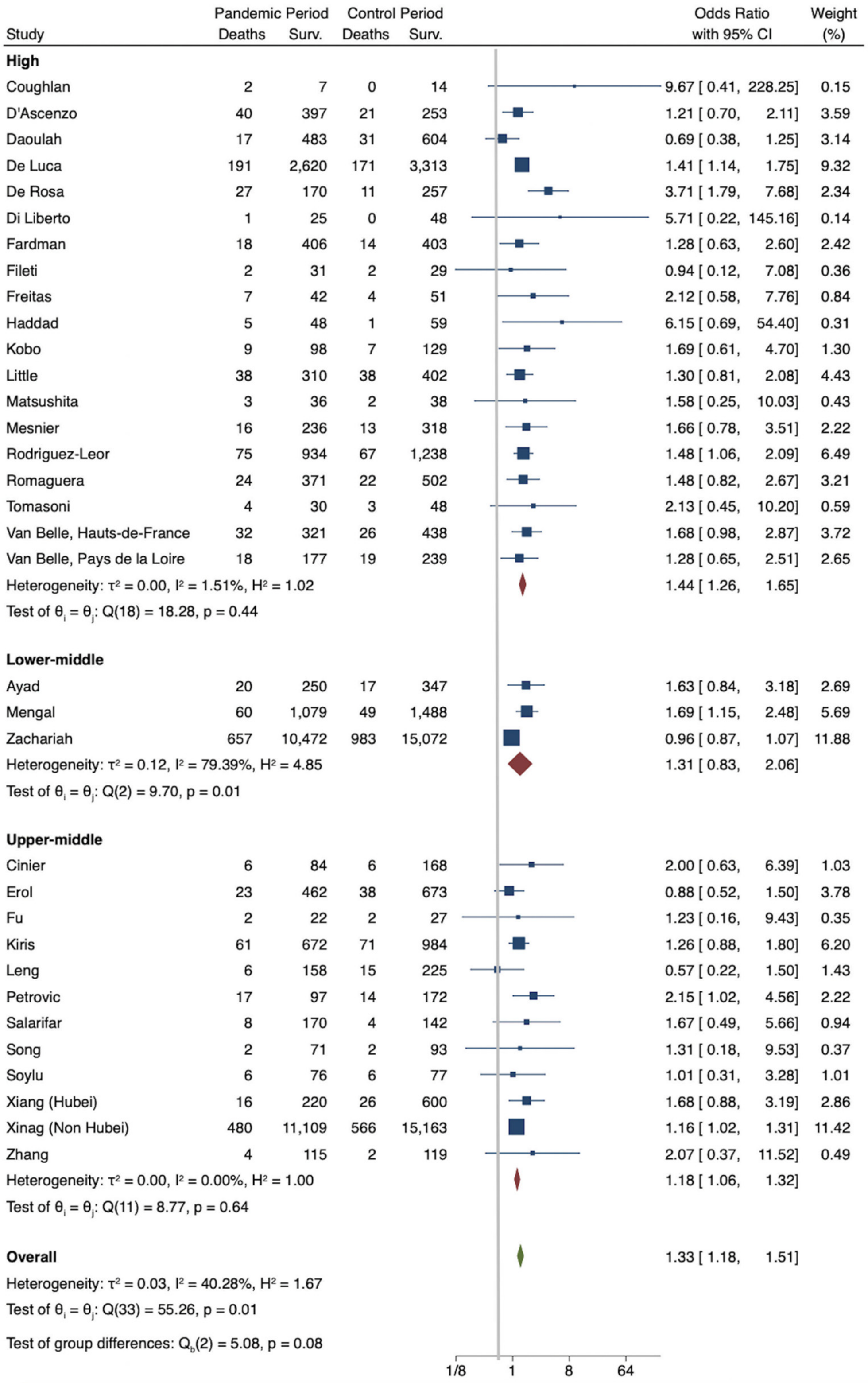

Figure S6. STEMI. Mortality subgroup analysis (ORs, 95% CI) according to income levels.

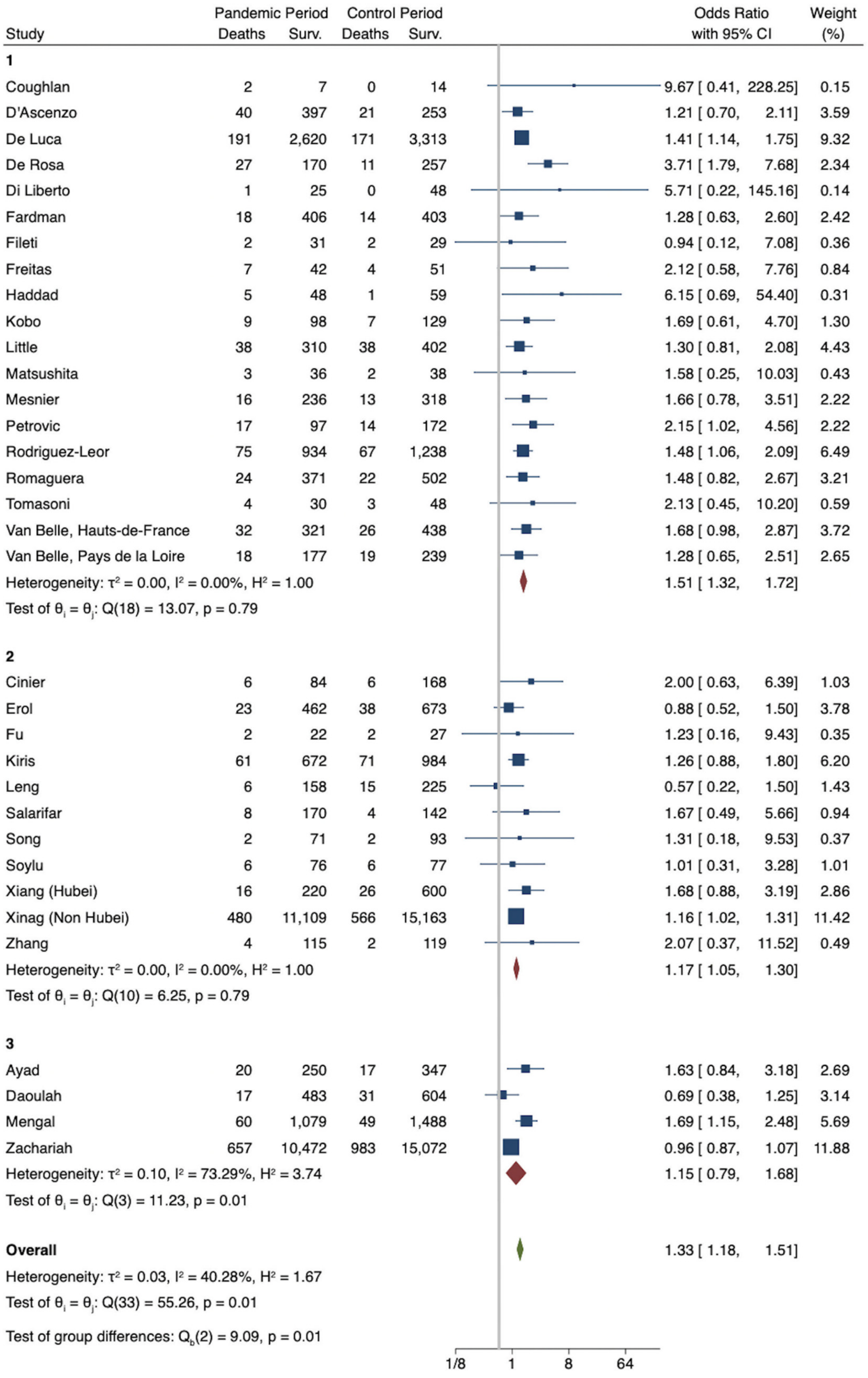

Figure S7. STEMI. Mortality subgroup analysis (ORs, 95% CI) according to data quality.

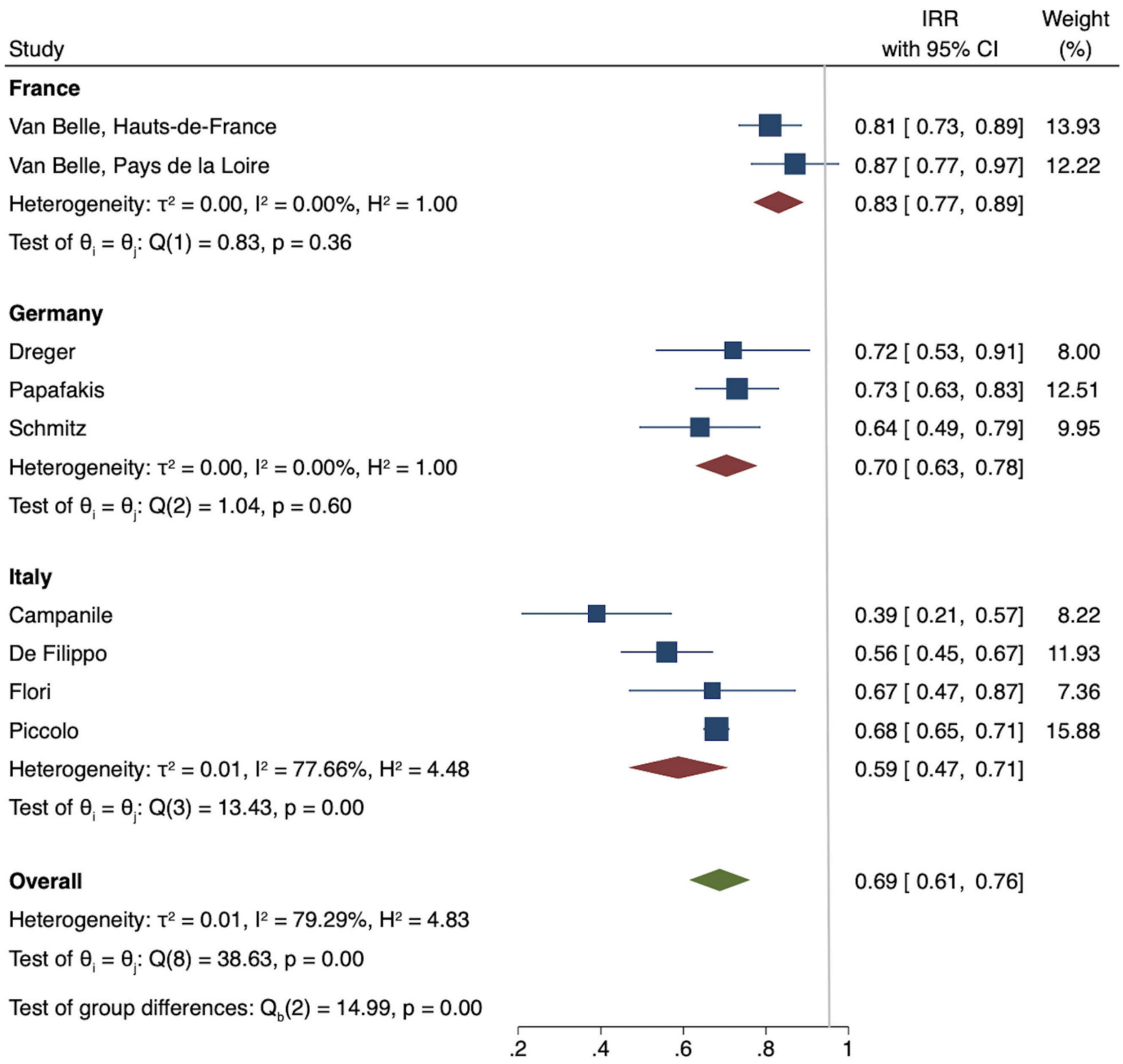

Figure S8. NSTEMI. Hospital admission subgroup analysis (ORs, 95% CI) according to country.

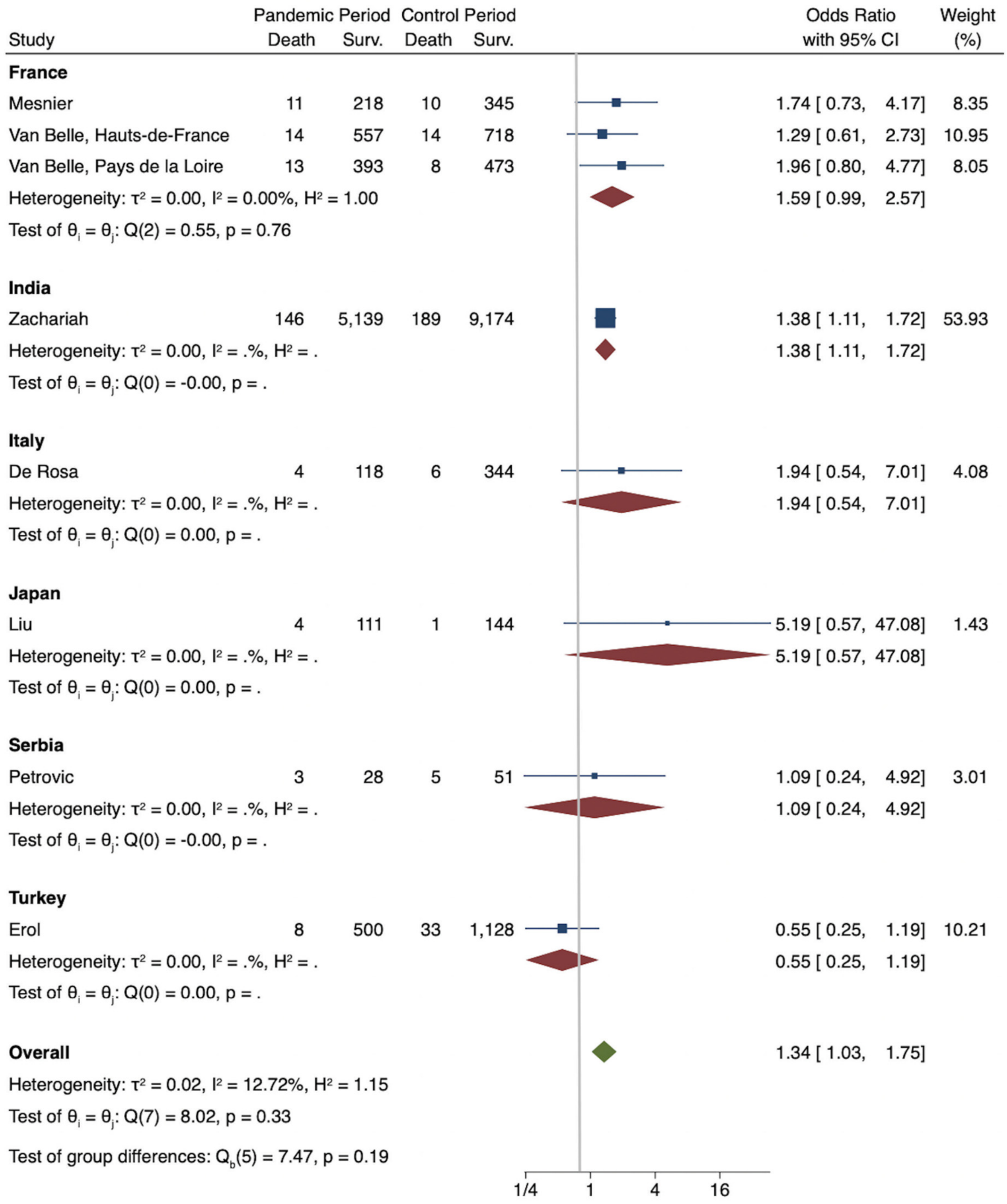

Figure S9. NSTEMI. Mortality subgroup analysis (ORs, 95% CI) according to country.

Table S1. Research strategy.

PUBMED	
(stemi incidence) OR (stemi hospitalization)) AND (("COVID-19"[Mesh]) AND ("ST Elevation Myocardial Infarction"[Mesh])) ((("ST Elevation Myocardial Infarction"[MeSH Terms] OR ("st"[All Fields] AND "elevation"[All Fields] AND "myocardial"[All Fields] AND "infarction"[All Fields]) OR "ST Elevation Myocardial Infarction"[All Fields] OR "stemi"[All Fields] OR "stemis"[All Fields]) AND ("epidemiology"[MeSH Subheading] OR "epidemiology"[All Fields] OR "incidence"[All Fields] OR "incidence"[MeSH Terms] OR "incidences"[All Fields] OR "incident"[All Fields] OR "incidents"[All Fields])) OR (("ST Elevation Myocardial Infarction"[MeSH Terms] OR ("st"[All Fields] AND "elevation"[All Fields] AND "myocardial"[All Fields] AND "infarction"[All Fields]) OR "ST Elevation Myocardial Infarction"[All Fields] OR "stemi"[All Fields] OR "stemis"[All Fields]) AND ("hospital s"[All Fields] OR "hospitalisation"[All Fields] OR "hospitalization"[MeSH Terms] OR "hospitalization"[All Fields] OR "hospitalising"[All Fields] OR "hospitality"[All Fields] OR "hospitalisations"[All Fields] OR "hospitalised"[All Fields] OR "hospitalizations"[All Fields] OR "hospitalized"[All Fields] OR "hospitalize"[All Fields] OR "hospitalizing"[All Fields] OR "hospitals"[MeSH Terms] OR "hospitals"[All Fields] OR "hospital"[All Fields]))) AND ("COVID-19"[MeSH Terms] AND "ST Elevation Myocardial Infarction"[MeSH Terms]) COVID-19"[MeSH Terms] AND "ST Elevation Myocardial Infarction"[MeSH Terms]	251
((("Non-ST Elevated Myocardial Infarction"[MeSH Terms] OR ("non st"[All Fields] AND "elevated"[All Fields] AND "myocardial"[All Fields] AND "infarction"[All Fields]) OR "Non-ST Elevated Myocardial Infarction"[All Fields] OR "nstemi"[All Fields] OR "nstemis"[All Fields]) AND ("epidemiology"[MeSH Subheading] OR "epidemiology"[All Fields] OR "incidence"[All Fields] OR "incidence"[MeSH Terms] OR "incidences"[All Fields] OR "incident"[All Fields] OR "incidents"[All Fields])) OR (("Non-ST Elevated Myocardial Infarction"[MeSH Terms] OR ("non st"[All Fields] AND "elevated"[All Fields] AND "myocardial"[All Fields] AND "infarction"[All Fields]) OR "Non-ST Elevated Myocardial Infarction"[All Fields] OR "nstemi"[All Fields] OR "nstemis"[All Fields]) AND ("hospital s"[All Fields] OR "hospitalisation"[All Fields] OR "hospitalization"[MeSH Terms] OR "hospitalization"[All Fields] OR "hospitalising"[All Fields] OR "hospitality"[All Fields] OR "hospitalisations"[All Fields] OR "hospitalised"[All Fields] OR "hospitalizations"[All Fields] OR "hospitalized"[All Fields] OR "hospitalize"[All Fields] OR "hospitalizing"[All Fields] OR "hospitals"[MeSH Terms] OR "hospitals"[All Fields] OR "hospital"[All Fields]))) AND "COVID-19"[MeSH Terms] AND "Non-ST Elevated Myocardial Infarction"[MeSH Terms]	31
((("Acute Coronary Syndrome"[MeSH Terms] OR ("acute"[All Fields] AND "coronary"[All Fields] AND "syndrome"[All Fields]) OR "Acute Coronary Syndrome"[All Fields]) AND ("epidemiology"[MeSH Subheading] OR "epidemiology"[All Fields] OR "incidence"[All Fields] OR "incidence"[MeSH Terms] OR "incidences"[All Fields] OR "incident"[All Fields] OR "incidents"[All Fields])) OR (("Acute Coronary Syndrome"[MeSH Terms] OR ("acute"[All Fields] AND "coronary"[All Fields] AND "syndrome"[All Fields]) OR "Acute Coronary Syndrome"[All Fields]) AND ("hospital s"[All Fields] OR "hospitalisation"[All Fields] OR "hospitalization"[MeSH Terms] OR "hospitalization"[All Fields] OR "hospitalising"[All Fields] OR "hospitality"[All Fields] OR "hospitalisations"[All Fields] OR "hospitalised"[All Fields] OR "hospitalizations"[All Fields] OR "hospitalized"[All Fields] OR "hospitalize"[All Fields] OR "hospitalizing"[All Fields] OR "hospitals"[MeSH Terms] OR "hospitals"[All Fields] OR "hospital"[All Fields]))) AND "COVID-19"[MeSH Terms] AND "Acute Coronary Syndrome"[MeSH Terms]	137
((("ST Elevation Myocardial Infarction"[MeSH Terms] OR ("st"[All Fields] AND "elevation"[All Fields] AND "myocardial"[All Fields] AND "infarction"[All Fields]) OR "ST Elevation Myocardial Infarction"[All Fields] OR "stemi"[All Fields] OR "stemis"[All Fields]) AND ("epidemiology"[MeSH Subheading] OR "epidemiology"[All Fields] OR "incidence"[All Fields] OR "incidence"[MeSH Terms] OR "incidences"[All Fields] OR "incident"[All Fields] OR "incidents"[All Fields])) OR (("ST Elevation Myocardial Infarction"[MeSH Terms] OR ("st"[All Fields] AND "elevation"[All Fields] AND "myocardial"[All Fields] AND "infarction"[All Fields]) OR "ST Elevation Myocardial Infarction"[All Fields] OR "stemi"[All Fields] OR "stemis"[All Fields]) AND ("hospital s"[All Fields] OR "hospitalisation"[All Fields] OR "hospitalization"[MeSH Terms] OR "hospitalization"[All Fields] OR "hospitalising"[All Fields] OR "hospitality"[All Fields] OR "hospitalisations"[All Fields] OR "hospitalised"[All Fields] OR "hospitalizations"[All Fields] OR "hospitalized"[All Fields] OR "hospitalize"[All Fields] OR "hospitalizing"[All Fields] OR "hospitals"[MeSH Terms] OR "hospitals"[All Fields] OR "hospital"[All Fields]))) AND ("COVID-19"[MeSH Terms] AND "ST Elevation Myocardial Infarction"[MeSH Terms]) and Lockdown	26

(("Non-ST Elevated Myocardial Infarction"[MeSH Terms] OR ("non st"[All Fields] AND "elevated"[All Fields] AND "myocardial"[All Fields] AND "infarction"[All Fields]) OR "Non-ST Elevated Myocardial Infarction"[All Fields] OR "nstemi"[All Fields] OR "nstemis"[All Fields]) AND ("epidemiology"[MeSH Subheading] OR "epidemiology"[All Fields] OR "incidence"[All Fields] OR "incidence"[MeSH Terms] OR "incidences"[All Fields] OR "incident"[All Fields] OR "incidents"[All Fields])) OR (("Non-ST Elevated Myocardial Infarction"[MeSH Terms] OR ("non st"[All Fields] AND "elevated"[All Fields] AND "myocardial"[All Fields] AND "infarction"[All Fields]) OR "Non-ST Elevated Myocardial Infarction"[All Fields] OR "nstemi"[All Fields] OR "nstemis"[All Fields]) AND ("hospital s"[All Fields] OR "hospitalisation"[All Fields] OR "hospitalization"[MeSH Terms] OR "hospitalization"[All Fields] OR "hospitalising"[All Fields] OR "hospitality"[All Fields] OR "hospitalisations"[All Fields] OR "hospitalised"[All Fields] OR "hospitalizations"[All Fields] OR "hospitalized"[All Fields] OR "hospitalize"[All Fields] OR "hospitalizing"[All Fields] OR "hospitals"[MeSH Terms] OR "hospitals"[All Fields] OR "hospital"[All Fields]))) AND "COVID-19"[MeSH Terms] AND "Non-ST Elevated Myocardial Infarction"[MeSH Terms] AND lockdown	8
((("Acute Coronary Syndrome"[MeSH Terms] OR ("acute"[All Fields] AND "coronary"[All Fields] AND "syndrome"[All Fields]) OR "Acute Coronary Syndrome"[All Fields]) AND ("epidemiology"[MeSH Subheading] OR "epidemiology"[All Fields] OR "incidence"[All Fields] OR "incidence"[MeSH Terms] OR "incidences"[All Fields] OR "incident"[All Fields] OR "incidents"[All Fields])) OR (("Acute Coronary Syndrome"[MeSH Terms] OR ("acute"[All Fields] AND "coronary"[All Fields] AND "syndrome"[All Fields]) OR "Acute Coronary Syndrome"[All Fields]) AND ("hospital s"[All Fields] OR "hospitalisation"[All Fields] OR "hospitalization"[MeSH Terms] OR "hospitalization"[All Fields] OR "hospitalising"[All Fields] OR "hospitality"[All Fields] OR "hospitalisations"[All Fields] OR "hospitalised"[All Fields] OR "hospitalizations"[All Fields] OR "hospitalized"[All Fields] OR "hospitalize"[All Fields] OR "hospitalizing"[All Fields] OR "hospitals"[MeSH Terms] OR "hospitals"[All Fields] OR "hospital"[All Fields]))) AND "COVID-19"[MeSH Terms] AND "Acute Coronary Syndrome"[MeSH Terms] and lockdown	**16**
**TOT PUBMED**	**469**
**SCOPUS**	
TITLE-ABS-KEY (stemi) OR TITLE-ABS-KEY-AUTH (st AND elevation AND myocardial AND infarction) AND TITLE-ABS-KEY (incidence) AND TITLE-ABS-KEY (hospitalization) AND TITLE-ABS-KEY (covid-19))	60
(TITLE-ABS-KEY (stemi) OR TITLE-ABS-KEY (st AND elevation AND myocardial AND infarction) AND TITLE-ABS-KEY (incidence) AND TITLE-ABS-KEY (hospitalization) AND TITLE-ABS-KEY (covid-19) AND TITLE-ABS-KEY (lockdown)	22
TITLE-ABS-KEY (nstemi) OR TITLE-ABS-KEY (non-st AND elevation AND myocardial AND infarction) AND TITLE-ABS-KEY (incidence) AND TITLE-ABS-KEY (hospitalization) AND TITLE-ABS-KEY (covid-19) AND TITLE-ABS-KEY (lockdown))	18
(TITLE-ABS-KEY (nstemi) OR TITLE-ABS-KEY (non-st AND elevated AND myocardial AND infarction) AND TITLE-ABS-KEY (incidence) AND TITLE-ABS-KEY (hospitalization) AND TITLE-ABS-KEY (covid-19))	9
TITLE-ABS-KEY (acs) OR TITLE-ABS-KEY (acute AND coronary AND syndromes) AND TITLE-ABS-KEY (incidence) ANDTITLE-ABS-KEY (hospitalization) AND TITLE-ABS-KEY (covid-19))	90
TITLE-ABS-KEY (acs) OR TITLE-ABS-KEY (acute AND coronary AND syndromes) AND TITLE-ABS-KEY (incidence) ANDTITLE-ABS-KEY (hospitalization) AND TITLE-ABS-KEY (covid-19) AND TITLE-ABS-KEY (lockdown))	18
**TOT SCOPUS**	**217**
**SCIENCE DIRECT**	
STEMI OR ST Elevation Myocardial Infarction AND incidence AND hospitalization AND covid-19	1989
STEMI OR ST Elevation Myocardial Infarction AND incidence AND hospitalization AND covid-19 AND LOCKDOWN	1142
NSTEMI OR non-ST Elevation Myocardial Infarction AND incidence AND hospitalization AND covid-19	1458
NSTEMI OR non-ST Elevation Myocardial Infarction AND incidence AND hospitalization AND covid-19 AND LOCKDOWN	627
ACS OR Acute cororany syndromes AND incidence AND hospitalization AND covid-19	4011

ACS OR Acute cororany syndromes AND incidence AND hospitalization AND covid-19 AND LOCKDOWN	4011
**TOT SCIENCEDIRECT** Filters applied for each search:<br>Medicine and Dentistry and Nursing and Health Professions<br>Research articles, Review articles	**13238**
**EMBASE**	
Stemi or 'st segment elevation myocardial infarction'/exp AND hospitalization and incidence AND covid 19	2953
Stemi or 'st segment elevation myocardial infarction'/exp AND hospitalization and incidence AND covid 19 AND lockdown	2953
NStemi or 'non st segment elevation myocardial infarction'/exp AND hospitalization and incidence AND covid 19	2955
NStemi or 'non st segment elevation myocardial infarction'/exp AND hospitalization and incidence AND covid 19 AND lockdown	2955
acs or 'acute coronary syndrome'/exp OR 'acute coronary syndrome' " and COVID 19 and incidence and hospitalization	2902
" acs or 'acute coronary syndrome'/exp OR 'acute coronary syndrome' " and COVID 19 and incidence and hospitalization and lockdown"	2902
**TOT EMBASE**	**17620**
**COCHRANE DATABASE**	
(((covid OR covid 19 OR sars cov OR sars cov 2 OR coronavirus) AND (cardiovascular OR acute coronary syndrome* OR myocardial infarct* OR stemi OR st segment elevation myocardial infarction))):ti,ab,kw OR (ST Elevation Myocardial Infarction):ti,ab,kw AND (covid-19):ti,ab,kw	407
(((covid OR covid 19 OR sars cov OR sars cov 2 OR coronavirus) AND (cardiovascular OR acute coronary syndrome* OR myocardial infarct* OR nstemi OR non-st segment elevation myocardial infarction))):ti,ab,kw OR (ST Elevation Myocardial Infarction):ti,ab,kw AND (covid-19):ti,ab,kw	400
**TOT COCHRANE DATABASE**	807
**WEB OF SCIENCE**	
((covid OR covid 19 OR sars cov OR sars cov 2 OR coronavirus) AND (cardiovascular OR acute coronary syndrome* OR myocardial infarct* OR stemi OR st segment elevation myocardial infarction or NSTEMI or non- st segment elevation myocardial infarction)) and hospitalization AND incidence	78
**TOT WEB OF SCIENCE**	**78**

Table S2. PRISMA checklist.

Section and Topic	Item #	Checklist Item	Location Where Item Is Reported
**TITLE**			
Title	1	Identify the report as a systematic review.	1
**ABSTRACT**			
Abstract	2	See PRISMA 2020 for Abstracts checklist.	
**INTRODUCTION**			
Rationale	3	Describe the rationale for the review in the context of existing knowledge.	2
Objectives	4	Provide an explicit statement of the objective(s) or question(s) the review addresses.	2
**METHODS**			
Eligibility criteria	5	Specify the inclusion and exclusion criteria for the review and how studies were grouped for the syntheses.	6–7
Information sources	6	Specify all databases, registers, websites, organisations, reference lists and other sources searched or consulted to identify studies. Specify the date when each source was last searched or consulted.	6–7
Search strategy	7	Present the full search strategies for all databases, registers and websites, including any filters and limits used.	6–7<br>suppl Table S1
Selection process	8	Specify the methods used to decide whether a study met the inclusion criteria of the review, including how many reviewers screened each record and each report retrieved, whether they worked independently and, if applicable, details of automation tools used in the process.	6–7
Data collection process	9	Specify the methods used to collect data from reports, including how many reviewers collected data from each report, whether they worked independently, any processes for obtaining or confirming data from study investigators and, if applicable, details of automation tools used in the process.	6–7
Data items	10a	List and define all outcomes for which data were sought. Specify whether all results that were compatible with each outcome domain in each study were sought (e.g., for all measures, time points, analyses) and, if not, the methods used to decide which results to collect.	6–7<br>suppl Table S3
	10b	List and define all other variables for which data were sought (e.g., participant and intervention characteristics, funding sources). Describe any assumptions made about any missing or unclear information.	6–7
Study risk of bias assessment	11	Specify the methods used to assess risk of bias in the included studies, including details of the tool(s) used, how many reviewers assessed each study and whether they worked independently and, if applicable, details of automation tools used in the process.	6–7<br>suppl Table S3
Effect measures	12	Specify for each outcome the effect measure(s) (e.g., risk ratio, mean difference) used in the synthesis or presentation of results.	6–7
Synthesis methods	13a	Describe the processes used to decide which studies were eligible for each synthesis (e.g., tabulating the study intervention characteristics and comparing against the planned groups for each synthesis (item #5)).	6–7
	13b	Describe any methods required to prepare the data for presentation or synthesis, such as handling of missing summary statistics or data conversions.	6–7
	13c	Describe any methods used to tabulate or visually display results of individual studies and syntheses.	6–7

	13d	Describe any methods used to synthesize results and provide a rationale for the choice(s). If a meta-analysis was performed, describe the model(s), method(s) to identify the presence and extent of statistical heterogeneity, and software package(s) used.	6–7
	13e	Describe any methods used to explore possible causes of heterogeneity among study results (e.g., subgroup analysis, meta-regression).	6–7
	13f	Describe any sensitivity analyses conducted to assess robustness of the synthesized results.	6–7
Reporting bias assessment	14	Describe any methods used to assess risk of bias due to missing results in a synthesis (arising from reporting biases).	6–7
Certainty assessment	15	Describe any methods used to assess certainty (or confidence) in the body of evidence for an outcome.	6–7
**RESULTS**			
Study selection	16a	Describe the results of the search and selection process, from the number of records identified in the search to the number of studies included in the review, ideally using a flow diagram.	8–11
	16b	Cite studies that might appear to meet the inclusion criteria but which were excluded, and explain why they were excluded.	8–11
Study characteristics	17	Cite each included study and present its characteristics.	8–11
Risk of bias in studies	18	Present assessments of risk of bias for each included study.	suppl Table S3
Results of individual studies	19	For all outcomes, present for each study: (a) summary statistics for each group (where appropriate) and (b) an effect estimate and its precision (e.g., confidence/credible interval), ideally using structured tables or plots.	8–11 Figures S1–S5; suppl Figures S2–S9
Results of syntheses	20a	For each synthesis, briefly summarize the characteristics and risk of bias among contributing studies.	8–11
	20b	Present results of all statistical syntheses conducted. If a meta-analysis was performed, present for each the summary estimate and its precision (e.g., confidence/credible interval) and measures of statistical heterogeneity. If comparing groups, describe the direction of the effect.	
	20c	Present results of all investigations of possible causes of heterogeneity among study results.	suppl Figures S2–S9
	20d	Present results of all sensitivity analyses conducted to assess the robustness of the synthesized results.	
Reporting biases	21	Present assessments of risk of bias due to missing results (arising from reporting biases) for each synthesis assessed.	suppl Table S3
Certainty of evidence	22	Present assessments of certainty (or confidence) in the body of evidence for each outcome assessed.	8–11 Figures 1–5; suppl Figures S2–S9
**DISCUSSION**			
Discussion	23a	Provide a general interpretation of the results in the context of other evidence.	11–15
	23b	Discuss any limitations of the evidence included in the review.	11–15
	23c	Discuss any limitations of the review processes used.	11–15
	23d	Discuss implications of the results for practice, policy and future research.	11–15
**OTHER INFORMATION**			
Registration and protocol	24a	Provide registration information for the review, including register name and registration number, or state that the review was not registered.	not registered
	24b	Indicate where the review protocol can be accessed, or state that a protocol was not prepared.	not protocol
	24c	Describe and explain any amendments to information provided at registration or in the protocol.	-
Support	25	Describe sources of financial or non-financial support for the review, and the role of the funders or sponsors in the review.	-

Competing interests	26	Declare any competing interests of review authors.	-
Availability of data, code and other materials	27	Report which of the following are publicly available and where they can be found: template data collection forms; data extracted from included studies; data used for all analyses; analytic code; any other materials used in the review.	-

Table S3. Included studies, calendar weeks, quality assessment and outcomes.

**Pandemic Period vs. Control Period**																		
**Study id**	**Calendar Weeks**	**Country**	**Quality Assessment**	**STEMI**												**NSTEMI**		
			**Newcastle–Ottawa**	**AD**	**ADM IRR**	**MO**	**FM**	**DTB**	**CS**	**Trop B**	**Trop P**	**EF(%) AD**	**EF (%) DIS**	**LS**	**MC**	**AD**	**ADM IRR**	**MO**
**Abdelaziz [59]**	1–31/03 2020<br>1–31/03 2019	UK	6			x	x		x	x				x				
**Ayad [30]**	1/02–31/10 2020<br>1/02–31/10 2019	Egypt	6			x	x		x	x				x				
**Boeddinghaus [60]**	Jan–Apr 2020<br>Jan Apr 2019	Switzerland	7				x	x		x	x		x					
**Braiteh [70]**	March–Apr 2020<br>March–Apr 2019	USA	6	x												x		
**Bryndza [56]**	March–Apr 2020<br>March–Apr 2019	Poland	6												x			
**Cammalleri [61]**	March 2020<br>March 2019	Italy	7				x	x		x	x	x	x	x	x			
**Campanile [22]**	10/03–9/04 2020<br>8/02-9/03 2020	Italy	7		x												x	
**Cinier [31]**	05/03–06/04 2020<br>05/03– 06/04 2019	Turkey	6			x	x	x					x					
**Clifford [57]**	27/03–17/04 2020<br>27/03–17/04 2019	Canada	7				x							x				

**Coughlan [32]**	27/03–17/04 2020<br>27/03–17/04 2029	Ireland	7			x	x											
**D'Ascenzo [15]**	20/03–3/05 2020<br>20/03–3/05 2019	Italy	8	x		x			x						x	x		
**Daoullah [33]**	01/01–30/04 2020<br>01/01–30/04 2019	Saudi Arabia	8			x			x	x				x				
**De Filippo [4]**	20/03–31/03 2020<br>20/03–31/03 2019	Italy	8		x												x	
**De Luca [34]**	March 2020<br>March 2019	Multinational	8			x		x	x							x		
**De Rosa [7]**	12–19 March2020<br>12–19 March2019	Italy	7	x		x												x
**Di Liberto [35]**	1/03–24/04 2020<br>1/03–24/04 2019	Italy	6			x												
**Dreger [23]**	Calendar weeks 2–21 2020<br>Calendar weeks 2–21 2019	Germany	6		x												x	
**Erol [36]**	April 17– May 02 2020<br>1–15 Nov 02 2018	Turkey	8			x	x	x	x					x		x		x
**Fardman [24]**	9–03/4–04 2020<br>9–03/4–04 2018	Israel	7		x	x	x	x	x			x		x	x			
**Fileti [37]**	10/04–10/05 2020<br>10/04–10/05 2019	Italy	7	x		x							x	x			x	

**Flori [68]**	20–02/15–04 2020<br>20–02/15–04 2019	Italy	6														x	
**Freitas [38]**	March–Apr 2020<br>March–Apr 2019	Portugal	7			x							x					
**Fu [39]**	20–01/20–04 2020<br>20–01/20–04 2019	China	6			x	x	x										
**Haddad [40]**	Mid March/Mid May 2020<br>Mid March/Mid May 2019	Israel	6			x	x		x			x			x			
**Kiris [41]**	11–03/15–05 2020<br>11–03/15–05 2019	Turkey	8			x	x		x			x						
**Kobo [42]**	20–03/30–04 2020<br>20–03/30–04 2019	Israel	8			x	x	x		x	x			x				
**Leng [43]**	23–01/30–04 2020<br>23–01/30–04 2019	China	6			x		x	x			x		x	x			
**Li [62]**	01/02–30/04 2020<br>01/02 –30/04 2019	China	6				x	x										
**Little [44]**	1–03/30–04 2020<br>1–03/30–04 2019	UK	6	x		x	x	x						x				
**Liu [69]**	1/02–31/03 2020<br>1/02–31–03 2019	China	6															x
**Mathusita [45]**	01/03–20/04 2020<br>01/03–20/04 2019	France	7	x		x				x	x	x		x				
**Mengal [46]**	1–03/30–04 2020<br>1–03/30–04 2019	Pakistan	7			x						x						

**Mesnier [25]**	16/03–12/04 2020<br>17/02–11/04 2020	France	8		x	x	x										x	
**Metzler [5]**	Calendar week 12–13 2020<br>Calendar week 10–11 2020	Austria	5	x														
**Nef [13]**	23/03–26/04 2020<br>23/03–26/04 2019	Germany	6	x				x								x		
**Papafakis [26]**	2–03/12–04 2020<br>2–03/12–04 2019	Greece	7		x												x	
**Perrin [58]**	13–03/30/04 2020<br>13–03/30/04 2019	Switzerland	7				x				x			x				
**Petrovic [47]**	16/03–06/05 2020<br>16/03–06/05 2019	Serbia	8	x		x		x					x	x		x		x
**Piccolo [27]**	01/03–26/03 2020<br>30/01–29/02 2020	Italy	7		x												x	
**Primessnig [72]**	01/01–30/04 2020<br>01/01–30/04 2020	Germany	6	x												x		
**Rangashamaiah [67]**	23/03–05/05 2020<br>08/02–22/03 2020	India	5									x						
**Rodriguez-Leor [48]**	16/03–16/04 2020<br>1–30/04 2019	Spain	7			x	x		x						x			
**Romaguera [28]**	1-03/19–04 2020<br>1–03/19–04 2019	Spain	6		X	x		x										
**Salarifar [49]**	29/02–30/04 2020<br>29/02–30/04 2019	Iran	8			x	x	x										
**Schmitz [29]**	16/03–19/04 2020<br>10/02–13/03 2020	Germany	8		x												x	

**Simoni [73]**	09/03–30/04 2020<br>09/03–30/04 2019	Albania	7	x												x		
**Secco [63]**	March 2020<br>March 2019	Italy	7	x		x												
**Siudak [64]**	13/03–13/05 2020<br>13/03–13/05 2019	Poland	7	x			x									x		
**Solomon [74]**	04/03–14/04 2020<br>04/03–14/04 2019	USA			x											x		
**Song [50]**	24-01/31–03 2020<br>24–01/31–03 2019	China	7			x			x	x	x	x			x			
**Soylu [51]**	11/03 2020 –<br>15/05 2020<br>13/01– 10/03 2020	Turkey	7			x	x	x						x				
**Tomasoni [52]**	21/02–10/04 2020<br>03/01–20/02 2020	Italy	8			x	x			x	x			x	x			
**Trabattoni [65]**	08/03–10/04 2020<br>08/03–10/04 2019	Italy	6				x											
**Van Belle [14]**	01/01–17/01 2020<br>01/01 –17/01 2019	France	8	x	x	x										x	x	x
**Weissman [66]**	March–Apr 2020<br>March–Apr 2019	Israel	5				x											
**Xiang [53]**	24/01–27/02 2020<br>27–12 2019 23/01<br>2020	China	7			x	x	x	x									
**Zachariah [54]**	15/03–15/06 2020<br>15/03–15/06 2019	India	7			x		x										x

**Zhang [55]**	1–01/31–03 2020<br>1–01/31–03 2019	China	6			x	x	x										
**Zeymer [71]**	23/03–26/04 2020<br>23/03–26/04 2020	Germany	6	x												x		
**Pandemic period vs. early post-pandemic period**																		
**Study id**	**Calendar Weeks**	**Country**	**Quality Assessment**	**STEMI**												**NSTEMI**		
			**Newcastle–Ottawa**	**ADM**	**ADM IRR**	**MO**	**FM**	**DTB**	**CS**	**Trop B**	**Trop P**	**EF(%)AD**	**EF (%)DIS**	**LO**	**MC**	**ADM**	**ADM IRR**	**MO**
**Campanile [22]**	10/03–9/04 2020<br>18/05–17/06 2020	Italy	8		x												x	
**Rognoni [12]**	20/02.034/05 2020<br>4/05–12/07 2020	Italy	8		x												x	
**Schmitz [29]**	16/03–19/04 2020<br>20/04–21/05 2020	Germany	8		x												x	

LEGEND: ADM: number of admission; ADM IRR: admission incidence rate ratio; MO: mortality; FM: first medical contact; DTB: door to ballon time; CD: cardiogenic shock; Tropo B: troponine at baseline; Tropo P: Troponine peack; EF (%) AD ejection fraction at admission; EF (%) DIS ejection fraction at discharge; LS: length of stay; MC: mechanincal complication.

Table S4. References of reports excluded with reasons.

	**No outcome of Interest/No Data**
**1**	de Koning ER, Boogers MJ, Bosch J, de Visser M, Schalij MJ, Beeres SLMA. Emergency medical services evaluations for chest pain during first COVID-19 lockdown in Hollands-Midden, the Netherlands. Neth Heart J. 2021 Apr;29(4):224-229. doi: 10.1007/s12471-021-01545-y. Epub 2021 Feb 18. PMID: 33599968; PMCID: PMC7890775.
**2**	Gramegna M, Baldetti L, Beneduce A, Pannone L, Falasconi G, Calvo F, Pazzanese V, Sacchi S, Pagnesi M, Moroni F, Ajello S, Melisurgo G, Agricola E, Camici PG, Scandroglio AM, Landoni G, Ciceri F, Zangrillo A, Cappelletti AM. ST-Segment-Elevation Myocardial Infarction During COVID-19 Pandemic: Insights From a Regional Public Service Healthcare Hub. Circ Cardiovasc Interv. 2020 Aug;13(8):e009413. doi: 10.1161/CIRCINTERVENTIONS.120.009413. Epub 2020 Aug 14. PMID: 32791953.
**3**	Oikonomou E, Aznaouridis K, Barbetseas J, Charalambous G, Gastouniotis I, Fotopoulos V, Gkini KP, Katsivas A, Koudounis G, Koudounis P, Koutouzis M, Lamprinos D, Lazaris E, Lazaris E, Lazaros G, Marinos G, Platogiannis N, Platogiannis D, Siasos G, Terentes-Printzios D, Theodoropoulou A, Theofilis P, Toutouzas K, Tsalamandris S, Tsiafoutis I, Vavouranakis M, Vogiatzi G, Zografos T, Baka E, Tousoulis D, Vlachopoulos C. Hospital attendance and admission trends for cardiac diseases during the COVID-19 outbreak and lockdown in Greece. Public Health. 2020 Oct;187:115-119. doi: 10.1016/j.puhe.2020.08.007. Epub 2020 Aug 18. PMID: 32949881; PMCID: PMC7434308.
**4**	Rangé G, Hakim R, Beygui F, Angoulvant D, Marcollet P, Godin M, Deballon R, Bonnet P, Fichaux O, Barbey C, Viallard L, Lesault PF, Durand E, Boiffard E, Dutheil G, Collet JP, Benamer H, Commeau P, Montalescot G, Koning R, Motreff P. Incidence, delays, and outcomes of STEMI during COVID-19 outbreak: Analysis from the France PCI registry. J Am Coll Emerg Physicians Open. 2020 Nov 23;1(6):1168–76. doi: 10.1002/emp2.12325. Epub ahead of print. PMID: 33363285; PMCID: PMC7753646.
**5**	Toniolo M, Negri F, Antonutti M, Masè M, Facchin D. Unpredictable Fall of Severe Emergent Cardiovascular Diseases Hospital Admissions During the COVID-19 Pandemic: Experience of a Single Large Center in Northern Italy. J Am Heart Assoc. 2020 Jul 7;9(13):e017122. doi: 10.1161/JAHA.120.017122. Epub 2020 May 22. PMID: 32441548; PMCID: PMC7670496.
**6**	Huet F, Prieur C, Schurtz G, Gerbaud E, Manzo-Silberman S, Vanzetto G, Elbaz M, Tea V, Mercier G, Lattuca B, Duflos C, Roubille F. One train may hide another: Acute cardiovascular diseases could be neglected because of the COVID-19 pandemic. Arch Cardiovasc Dis. 2020 May;113(5):303-307. doi: 10.1016/j.acvd.2020.04.002. Epub 2020 Apr 28. PMID: 32362433; PMCID: PMC7186196.
**7**	Lantelme P, Couray Targe S, Metral P, Bochaton T, Ranc S, Le Bourhis Zaimi M, Le Coanet A, Courand PY, Harbaoui B. Worrying decrease in hospital admissions for myocardial infarction during the COVID-19 pandemic. Arch Cardiovasc Dis. 2020 Jun-Jul;113(6-7):443-447. doi: 10.1016/j.acvd.2020.06.001. Epub 2020 Jun 25. PMID: 32636131; PMCID: PMC7316064.
**8**	Zitelny E, Newman N, Zhao D. STEMI during the COVID-19 Pandemic - An Evaluation of Incidence. Cardiovasc Pathol. 2020 Sep-Oct;48:107232. doi: 10.1016/j.carpath.2020.107232. Epub 2020 May 1. PMID: 32454398; PMCID: PMC7194046.
**9**	Kiblboeck D, Kellermair J, Siostrzonek P, Steinwender C. Regional differences in hospital admissions for ST-elevation and non-ST-elevation myocardial infarctions during the Coronavirus disease-19 (COVID-19) pandemic in Austria. Wien Klin Wochenschr. 2020 Jul;132(13-14):362-364. doi: 10.1007/s00508-020-01698-7. Epub 2020 Jun 12. PMID: 32533442; PMCID: PMC7291188.
**10**	Kumar M, Tyagi N, Arora M. Management of ST elevation myocardial infarction (STEMI) with primary angioplasty in Covid 19 lockdown. Indian Heart J. 2020 Jul-Aug;72(4):325-326. doi: 10.1016/j.ihj.2020.06.014. Epub 2020 Jun 30. PMID: 32861396; PMCID: PMC7474101.

11	Versaci F, Scappaticci M, Calcagno S, Del Prete A, Romeo F, Peruzzi M, Cavarretta E, Frati G. ST-elevation myocardial infarction in the COVID-19 era. Minerva Cardiol Angiol. 2021 Feb;69(1):6-8. doi: 10.23736/S2724-5683.20.05343-8. Epub 2020 May 29. PMID: 32472994.
12	Rodriguez-Leor O, Cid Alvarez AB, Pérez de Prado A, Rossello X, Ojeda S, Serrador A, López-Palop R, Martin-Moreiras J, Rumoroso JR, Cequier A, Ibáñez B, Cruz-González I, Romaguera R, Moreno R. In-hospital outcomes of COVID-19 ST-elevation myocardial infarction patients. EuroIntervention. 2021 Apr 20;16(17):1426-1433. doi: 10.4244/EIJ-D-20-00935. PMID: 33164893.
13	Moreno R, Alonso JJ, Caballero R, Del Corral E, Elízaga J, Asenjo RM, Mena MJ, Alfonso F, Fernández-Ortiz A, Goicolea FJ, Botas J, Navarro F, Alegría-Barrero E, Ansede JC, de Sá EL, López-Sendón JL. Age and Gender influence on time of arrival for STEMI patients during Covid-19 pandemic. Am J Emerg Med. 2021 Apr;42:244-245. doi: 10.1016/j.ajem.2020.06.013. Epub 2020 Jun 6. PMID: 32540217; PMCID: PMC7274966.
14	Nan J, Meng S, Hu H, Jia R, Chen W, Li Q, Zhang T, Song K, Wang Y, Jin Z. Comparison of Clinical Outcomes in Patients with ST Elevation Myocardial Infarction with Percutaneous Coronary Intervention and the Use of a Telemedicine App Before and After the COVID-19 Pandemic at a Center in Beijing, China, from August 2019 to March 2020. Med Sci Monit. 2020 Sep 17;26:e927061. doi: 10.12659/MSM.927061. PMID: 32938901; PMCID: PMC7521072.
15	Oikonomou E, Aznaouridis K, Barbetseas J, Charalambous G, Gastouniotis I, Fotopoulos V, Gkini KP, Katsivas A, Koudounis G, Koudounis P, Koutouzis M, Lamprinos D, Lazaris E, Lazaris E, Lazaros G, Marinos G, Platogiannis N, Platogiannis D, Siasos G, Terentes-Printzios D, Theodoropoulou A, Theofilis P, Toutouzas K, Tsalamandris S, Tsiafoutis I, Vavouranakis M, Vogiatzi G, Zografos T, Baka E, Tousoulis D, Vlachopoulos C. Hospital attendance and admission trends for cardiac diseases during the COVID-19 outbreak and lockdown in Greece. Public Health. 2020 Oct;187:115-119. doi: 10.1016/j.puhe.2020.08.007. Epub 2020 Aug 18. PMID: 32949881; PMCID: PMC7434308.
16	Grave C, Gabet A, Puymirat E, Empana JP, Tuppin P, Danchin N, Olié V. Myocardial infarction throughout 1 year of the COVID-19 pandemic: French nationwide study of hospitalization rates, prognosis and 90-day mortality rates. Arch Cardiovasc Dis. 2021 Dec;114(12):768-780. doi: 10.1016/j.acvd.2021.10.008. Epub 2021 Nov 10. PMID: 34840126; PMCID: PMC8600551.
17	Gluckman TJ, Wilson MA, Chiu ST, Penny BW, Chepuri VB, Waggoner JW, Spinelli KJ. Case Rates, Treatment Approaches, and Outcomes in Acute Myocardial Infarction During the Coronavirus Disease 2019 Pandemic. JAMA Cardiol. 2020 Dec 1;5(12):1419-1424. doi: 10.1001/jamacardio.2020.3629. PMID: 32766756; PMCID: PMC7414426.
18	Rebollal-Leal F, Aldama-López G, Flores-Ríos X, Piñón-Esteban P, Salgado-Fernández J, Calviño-Santos R, Vázquez-González N, Vázquez-Rodríguez JM. Impact of COVID-19 outbreak and public lockdown on ST-segment elevation myocardial infarction care in Spain. Cardiol J. 2020;27(4):425-426. doi: 10.5603/CJ.a2020.0098. Epub 2020 Aug 4. PMID: 32748944; PMCID: PMC8015996.
19	Fabris E, Bessi R, De Bellis A, Gregorio C, Peratoner A, Lardieri G, Cominotto F, Vitrella G, Rakar S, Perkan A, Sinagra G. COVID-19 impact on ST-elevation myocardial infarction incidence rate in a Italian STEMI network: a U-shaped curve phenomenon. J Cardiovasc Med (Hagerstown). 2021 May 1;22(5):344-349. doi: 10.2459/JCM.0000000000001153. PMID: 33399345.
20	Piuhola J, Kerkelä R, Laine M, Andersen GØ, Ērglis A, Kumsārs I, Thuesen L, Sinisalo J, Niemelä M, Junttila MJ. Lower ST-elevation myocardial infarction incidence during COVID-19 epidemic in Northern Europe. Scand Cardiovasc J. 2020 Dec;54(6):358-360. doi: 10.1080/14017431.2020.1820563. Epub 2020 Sep 18. PMID: 32945201.
21	Frain K, Rathod KS, Tumi E, Chen Y, Hamshere S, Choudry F, Akhtar MM, Curtis M, Amersey R, Guttmann O, O'Mahony C, Jain A, Wragg A, Baumbach A, Mathur A, Jones DA, Rees P. The impact of the COVID-19 pandemic on the delivery of primary percutaneous coronary

	intervention in STEMI. Am J Cardiovasc Dis. 2021 Oct 25;11(5):647-658. PMID: 34849298; PMCID: PMC8611259.
**22**	Carugo S, Ferlini M, Castini D, Andreassi A, Guagliumi G, Metra M, Lombardi C, Cuccia C, Savonitto S, Piatti L, D'Urbano M, Lettieri C, Vandoni P, Lettino M, Marenzi G, Montorfano M, Zangrillo A, Castiglioni B, De Ponti R, Oltrona Visconti L. Management of acute coronary syndromes during the COVID-19 outbreak in Lombardy: The "macro-hub" experience. Int J Cardiol Heart Vasc. 2020 Dec;31:100662. doi: 10.1016/j.ijcha.2020.100662. Epub 2020 Nov 3. PMID: 33173807; PMCID: PMC7609053.
**23**	Kuitunen I, Ponkilainen VT, Launonen AP, Reito A, Hevonkorpi TP, Paloneva J, Mattila VM. The effect of national lockdown due to COVID-19 on emergency department visits. Scand J Trauma Resusc Emerg Med. 2020 Dec 4;28(1):114. doi: 10.1186/s13049-020-00810-0. PMID: 33276799; PMCID: PMC7716110.
**24**	Bouisset F, Deney A, Ferrières J, Panagides V, Becker M, Riviere N, Yvorel C, Commeau P, Adjedj J, Benamer H, Bonnet G, Cayla G; MODIF registry investigators. Mechanical complications in ST-elevation myocardial infarction: The impact of pre-hospital delay. Int J Cardiol. 2021 Dec 15;345:14-19. doi: 10.1016/j.ijcard.2021.10.020. Epub 2021 Oct 23. PMID: 34699868.
**25**	Vecchio S, Fileti L, Reggi A, Moschini C, Lorenzetti S, Rubboli A. Impatto della pandemia COVID-19 sui ricoveri per sindrome coronarica acuta: revisione della letteratura ed esperienza monocentrica [Impact of the COVID-19 pandemic on admissions for acute coronary syndrome: review of the literature and single-center experience]. G Ital Cardiol (Rome). 2020 Jul;21(7):502-508. Italian. doi: 10.1714/3386.33635. PMID: 32555565.
**26**	Rodríguez-Leor O., Cid-Álvarez B., Ojeda S., et al. Impacto de la pandemia de COVID-19 sobre la actividad asistencial en cardiología intervencionista en España. REC Interventional Cardiology. 2020;2:82–89
**27**	Natarajan MK, Wijeysundera HC, Oakes G, Cantor WJ, Miner SES, Welsford M, Cheskes S, Le May MR, Jeffrey J, Ko DT. Early Observations During the COVID-19 Pandemic in Cardiac Catheterization Procedures for ST-Elevation Myocardial Infarction Across Ontario. CJC Open. 2020 Nov;2(6):678-683. doi: 10.1016/j.cjco.2020.07.015. Epub 2020 Jul 23. PMID: 32838257; PMCID: PMC7376355.
	**Different Calendar periods and temporal asymmetry**
**1**	Chew NW, Sia CH, Wee HL, Benedict LJ, Rastogi S, Kojodjojo P, Chor WPD, Leong BS, Koh BC, Tam H, Quek LS, Sia WC, Saw KW, Tung BW, Ng ZZ, Ambhore A, Tay EL, Chan KH, Lee CH, Loh JP, Low AF, Chan MY, Yeo TC, Tan HC, Loh PH. Impact of the COVID-19 Pandemic on Door-to-Balloon Time for Primary Percutaneous Coronary Intervention - Results From the Singapore Western STEMI Network. Circ J. 2021 Jan 25;85(2):139-149. doi: 10.1253/circj.CJ-20-0800. Epub 2020 Nov 7. PMID: 33162491.
**2**	Hammad TA, Parikh M, Tashtish N, Lowry CM, Gorbey D, Forouzandeh F, Filby SJ, Wolf WM, Costa MA, Simon DI, Shishehbor MH. Impact of COVID-19 pandemic on ST-elevation myocardial infarction in a non-COVID-19 epicenter. Catheter Cardiovasc Interv. 2021 Feb 1;97(2):208-214. doi: 10.1002/ccd.28997. Epub 2020 Jun 1. PMID: 32478961; PMCID: PMC7300525.
**3**	Hannan EL, Wu Y, Cozzens K, Friedrich M, Tamis-Holland J, Jacobs AK, Ling FSK, King SB 3rd, Venditti FJ, Walford G, Berger PB, Kirtane AJ, Kamran M. Percutaneous Coronary Intervention for ST-Elevation Myocardial Infarction Before and During COVID in New York. Am J Cardiol. 2021 Mar 1;142:25-34. doi: 10.1016/j.amjcard.2020.11.033. Epub 2020 Dec 8. PMID: 33301770; PMCID: PMC7723434.
**4**	Kitahara S, Fujino M, Honda S, Asaumi Y, Kataoka Y, Otsuka F, Nakanishi M, Tahara Y, Ogata S, Onozuka D, Nishimura K, Fujita T, Tsujita K, Ogawa H, Noguchi T. COVID-19 pandemic is associated with mechanical complications in patients with ST-elevation myocardial infarction. Open Heart. 2021 Feb;8(1):e001497. doi: 10.1136/openhrt-2020-001497. PMID: 33547221; PMCID: PMC7871043.

5	Kwok CS, Gale CP, Kinnaird T, Curzen N, Ludman P, Kontopantelis E, Wu J, Denwood T, Fazal N, Deanfield J, de Belder MA, Mamas M. Impact of COVID-19 on percutaneous coronary intervention for ST-elevation myocardial infarction. Heart. 2020 Dec;106(23):1805-1811. doi: 10.1136/heartjnl-2020-317650. Epub 2020 Aug 31. PMID: 32868280.
6	Mafham MM, Spata E, Goldacre R, Gair D, Curnow P, Bray M, Hollings S, Roebuck C, Gale CP, Mamas MA, Deanfield JE, de Belder MA, Luescher TF, Denwood T, Landray MJ, Emberson JR, Collins R, Morris EJA, Casadei B, Baigent C. COVID-19 pandemic and admission rates for and management of acute coronary syndromes in England. Lancet. 2020 Aug 8;396(10248):381-389. doi: 10.1016/S0140-6736(20)31356-8. Epub 2020 Jul 14. PMID: 32679111; PMCID: PMC7429983
7	Arai R, Fukamachi D, Ebuchi Y, Migita S, Morikawa T, Monden M, Takei N, Tamaki T, Kojima K, Akutsu N, Murata N, Kitano D, Okumura Y. Impact of the COVID-19 outbreak on hospitalizations and outcomes in patients with acute myocardial infarction in a Japanese Single Center. Heart Vessels. 2021 Oct;36(10):1474-1483. doi: 10.1007/s00380-021-01835-w. Epub 2021 Mar 20. PMID: 33743048; PMCID: PMC7980755.
8	Mitra B, Mitchell RD, Cloud GC, Stub D, Nguyen M, Nanayakkara S, Miller JP, M O'Reilly G, Smit V, Cameron PA. Presentations of stroke and acute myocardial infarction in the first 28 days following the introduction of State of Emergency restrictions for COVID-19. Emerg Med Australas. 2020 Dec;32(6):1040-1045. doi: 10.1111/1742-6723.13621. Epub 2020 Sep 12. PMID: 32833297; PMCID: PMC7461453.
9	Scholz KH, Lengenfelder B, Thilo C, Jeron A, Stefanow S, Janssens U, Bauersachs J, Schulze PC, Winter KD, Schröder J, Vom Dahl J, von Beckerath N, Seidl K, Friede T, Meyer T. Impact of COVID-19 outbreak on regional STEMI care in Germany. Clin Res Cardiol. 2020 Dec;109(12):1511-1521. doi: 10.1007/s00392-020-01703-z. Epub 2020 Jul 16. PMID: 32676681; PMCID: PMC7364412.
10	Tam CF, Cheung KS, Lam S, Wong A, Yung A, Sze M, Lam YM, Chan C, Tsang TC, Tsui M, Tse HF, Siu CW. Impact of Coronavirus Disease 2019 (COVID-19) Outbreak on ST-Segment-Elevation Myocardial Infarction Care in Hong Kong, China. Circ Cardiovasc Qual Outcomes. 2020 Apr;13(4):e006631. doi: 10.1161/CIRCOUTCOMES.120.006631. Epub 2020 Mar 17. PMID: 32182131; PMCID: PMC7147280.
11	Chan DZ, Stewart RA, Kerr AJ, Dicker B, Kyle CV, Adamson PD, Devlin G, Edmond J, El-Jack S, Elliott JM, Fisher N, Flynn C, Lee M, Liao YB, Rhodes M, Scott T, Smith T, Stiles MK, Swain AH, Todd VF, Webster MW, Williams MJ, White HD, Somaratne JB. The impact of a national COVID-19 lockdown on acute coronary syndrome hospitalisations in New Zealand (ANZACS-QI 55). Lancet Reg Health West Pac. 2020 Dec;5:100056. doi: 10.1016/j.lanwpc.2020.100056. Epub 2020 Nov 20. PMID: 34173604; PMCID: PMC7677076.
12	Wienbergen H, Retzlaff T, Schmucker J, Marin LAM, Rühle S, Garstka D, Osteresch R, Fach A, Hambrecht R. Impact of COVID-19 Pandemic on Presentation and Outcome of Consecutive Patients Admitted to Hospital Due to ST-Elevation Myocardial Infarction. Am J Cardiol. 2021 Jul 15;151:10-14. doi: 10.1016/j.amjcard.2021.04.011. Epub 2021 Apr 27. PMID: 34049671; PMCID: PMC8075839.
13	Seiffert M, Brunner FJ, Remmel M, Thomalla G, Marschall U, L'Hoest H, Acar L, Debus ES, Blankenberg S, Gerloff C, Behrendt CA. Temporal trends in the presentation of cardiovascular and cerebrovascular emergencies during the COVID-19 pandemic in Germany: an analysis of health insurance claims. Clin Res Cardiol. 2020 Dec;109(12):1540-1548. doi: 10.1007/s00392-020-01723-9. Epub 2020 Aug 4. PMID: 32749558; PMCID: PMC7402080.
14	Garcia S, Albaghdadi MS, Meraj PM, Schmidt C, Garberich R, Jaffer FA, Dixon S, Rade JJ, Tannenbaum M, Chambers J, Huang PP, Henry TD. Reduction in ST-Segment Elevation Cardiac Catheterization Laboratory Activations in the United States During COVID-19 Pandemic. J Am Coll Cardiol. 2020 Jun 9;75(22):2871-2872. doi: 10.1016/j.jacc.2020.04.011. Epub 2020 Apr 10. PMID: 32283124; PMCID: PMC7151384.

**15**	Rollman JE, Kloner RA, Bosson N, Niemann JT, Gausche-Hill M, Williams M, Clare C, Tan W, Wang X, Shavelle DM, Rafique AM. Emergency Medical Services Responses to Out-of-Hospital Cardiac Arrest and Suspected ST-Segment-Elevation Myocardial Infarction During the COVID-19 Pandemic in Los Angeles County. J Am Heart Assoc. 2021 Jun 15;10(12):e019635. doi: 10.1161/JAHA.120.019635. Epub 2021 Jun 1. PMID: 34058862; PMCID: PMC8477893.
**16**	Wilson SJ, Connolly MJ, Elghamry Z, Cosgrove C, Firoozi S, Lim P, Sharma R, Spratt JC. Effect of the COVID-19 Pandemic on ST-Segment-Elevation Myocardial Infarction Presentations and In-Hospital Outcomes. Circ Cardiovasc Interv. 2020 Jul;13(7):e009438. doi: 10.1161/CIRCINTERVENTIONS.120.009438. PMID: 32600109.
**17**	Watanabe Y, Miyachi H, Mozawa K, Yamada K, Oka E, Shiomura R, Sugizaki Y, Matsuda J, Nakata J, Tara S, Tokita Y, Iwasaki YK, Yamamoto T, Takano H, Shimizu W. Impact of the COVID-19 Pandemic on ST-elevation Myocardial Infarction from a Single-center Experience in Tokyo. Intern Med. 2021 Dec 1;60(23):3693-3700. doi: 10.2169/internalmedicine.8220-21. Epub 2021 Sep 25. PMID: 34565777.
**18**	Wu J, Mamas M, Rashid M, Weston C, Hains J, Luescher T, de Belder MA, Deanfield JE, Gale CP. Patient response, treatments, and mortality for acute myocardial infarction during the COVID-19 pandemic. Eur Heart J Qual Care Clin Outcomes. 2021 May 3;7(3):238-246. doi: 10.1093/ehjqcco/qcaa062. PMID: 32730620; PMCID: PMC7454506.
**19**	Khot UN, Reimer AP, Brown A, Hustey FM, Hussain MS, Kapadia SR, Svensson LG. Impact of COVID-19 Pandemic on Critical Care Transfers for ST-Segment-Elevation Myocardial Infarction, Stroke, and Aortic Emergencies. Circ Cardiovasc Qual Outcomes. 2020 Aug;13(8):e006938. doi: 10.1161/CIRCOUTCOMES.120.006938. Epub 2020 Jun 11. PMID: 32524835.
	**Other reviews/meta-analysis**
**1**	Chew NWS, Ow ZGW, Teo VXY, Heng RRY, Ng CH, Lee CH, Low AF, Chan MY, Yeo TC, Tan HC, Loh PH. The Global Effect of the COVID-19 Pandemic on STEMI Care: A Systematic Review and Meta-analysis. Can J Cardiol. 2021 Sep;37(9):1450-1459. doi: 10.1016/j.cjca.2021.04.003. Epub 2021 Apr 20. PMID: 33848599; PMCID: PMC8056787.
**2**	Zhu Y, Xing W, Wang H, Song J, Sun Z, Li X. Characteristics of patients with ST-segment elevated myocardial infarction (STEMI) at the initial stage of the COVID-19 pandemic: a systematic review and meta-analysis. Infect Dis (Lond). 2021 Nov;53(11):865-875. doi: 10.1080/23744235.2021.1953131. Epub 2021 Jul 27. PMID: 34311652.
**3**	Sofi F, Dinu M, Reboldi G, Stracci F, Pedretti RFE, Valente S, Gensini G, Gibson CM, Ambrosio G. Worldwide differences of hospitalization for ST-segment elevation myocardial infarction during COVID-19: A systematic review and meta-analysis. Int J Cardiol. 2022 Jan 15;347:89-96. doi: 10.1016/j.ijcard.2021.10.156. Epub 2021 Nov 2. PMID: 34740717; PMCID: PMC8561779.
**4**	Baumhardt M, Dreyhaupt J, Winsauer C, Stuhler L, Thiessen K, Stephan T, Markovic S, Rottbauer W, Imhof A, Rattka M. The Effect of the Lockdown on Patients With Myocardial Infarction During the COVID-19 Pandemic–A Systematic Review and Meta-Analysis. Dtsch Arztebl Int. 2021 Jul 2;118(26):447-453. doi: 10.3238/arztebl.m2021.0253. PMID: 34114546; PMCID: PMC8383188.
**5**	Kamarullah W, Sabrina AP, Rocky MA, Gozali DR. Investigating the implications of COVID-19 outbreak on systems of care and outcomes of STEMI patients: A systematic review and meta-analysis. Indian Heart J. 2021 Jul-Aug;73(4):404-412. doi: 10.1016/j.ihj.2021.06.009. Epub 2021 Jun 25. PMID: 34474750; PMCID: PMC8257902.
**6**	Helal A, Shahin L, Abdelsalam M, Ibrahim M. Global effect of COVID-19 pandemic on the rate of acute coronary syndrome admissions: a comprehensive review of published literature. Open Heart. 2021 Jun;8(1):e001645. doi: 10.1136/openhrt-2021-001645. PMID: 34083389; PMCID: PMC8182753.

Table S5. Hospital admissions of STEMI and NSTEMI patients during COVID-19 pandemic in 2020 vs. corresponding pre-pandemic period.

	Pandemic Period			Control Period			Country	Income
**Author**	**Total**	**NSTEMI**	**STEMI**	**Total**	**NSTEMI**	**STEMI**		
Braiteh [70]	67	44	23	113	85	28	USA	High
Bryndza [56]	827	456	371	1055	599	456	Poland	High
D'Ascenzo [15]	691	254	437	675	289	386	Italy	High
De Rosa [7]	319	122	197	618	350	268	Italy	High
Erol [36]	991	506	485	1872	1161	711	Turkey	Upper–middle
Fileti [37]	57	38	34	67	58	36	Italy	High
Flori [69]	247	99	148	333	200	133	Italy	High
Zeymer [71]	144	98	46	238	189	49	Germany	High
Matsushita [45]	106	53	39	174	119	40	France	High
Mesnier [25]	481	229	252	686	355	331	France	High
Metzler [5]	288	129	159	437	242	195	Austria	High
Nef [13]	860	560	300	1061	750	311	Germany	High
Perrin [58]	45	12	33	140	74	66	Switzerland	High
Petrovic [47]	151	31	114	271	56	186	Serbia	Upper–middle
Primessnig [72]	51	26	25	96	61	35	Germany	High
Secco [65]	84	33	49	162	93	64	Italy	High
Simoni [73]	321	165	156	550	333	217	Albania	Upper–middle
Siudak [66]	5282	2862	2423	8427	4655	3772	Poland	High
Solomon [74]	453	78	370	628	526	102	USA	High
Trabattoni [65]	68	22	46	19	9	10	Italy	High
Van Belle, Hauts-de-France [14]	926	571	353	1196	732	464	France	High
Van Belle, Pays de la Loire [14]	601	406	195	739	481	258	France	High
Zachariah [54]	16414	5285	11129	25418	9363	16055	India	Lower–middle
**Total**	29474	12079	17384	44975	20780	24173		

Table S6. Meta-regressions results.

**STEMI**	**Age**	**Gender**	**DM**	**Smoking**	**Dyslipidemia**	**Hypertension**	**CKD**
Hospital admissions<br>OR	k = 6<br>*p* = 0.949	k = 5<br>*p* = 0.343	-	-	-	-	-
Overall mortality	k = 27<br>*p* = 0.066	k = 27<br>*p* = 0.011	k = 20<br>*p* = 0.081	k = 18<br>*p* = 0.468	k = 15<br>*p* = 0.818	k = 16<br>*p* = 0.145	k = 6<br>*p* = 0.767
Cardiogenic shock	k = 12<br>*p* = 0.830	k = 12<br>*p* = 0.152	k = 9<br>*p* = 0.652	k = 9<br>*p* = 0.438	k = 8<br>*p* = 0.379	k = 7<br>0.625	
LVEF at baseline	k = 9<br>*p* = 0.424	k = 7<br>*p* = 0.825	k = 7<br>*p* = 0.344	k = 7<br>*p* = 0.969	k = 6<br>*p* = 0.549	k = 6<br>*p* = 0.845	-
LVEF at discharge	k = 6<br>*p* = 0.579	k = 6<br>*p* = 0.362	k = 6<br>*p* = 0.290	k = 5<br>*p* = 0.211	k = 4<br>*p* = 0.615	k = 4<br>*p* = 0.780	-
Troponin at baseline	k = 9<br>*p* = 0.023	k = 8<br>*p* = 0.054	k = 8<br>*p* = 0.061	k = 7<br>*p* = 0.930	k = 7<br>*p* = 0.730	k = 7<br>*p* = 0.733	-
Troponin peak	k = 4<br>*p* = 0.632	-	-	-	-	-	-
Length of stay	k = 8<br>*p* = 0.043	k = 8<br>*p* = 0.045	k = 7<br>*p* = 0.107	k = 6<br>*p* = 0.272	-	k = 5<br>0 = 0.565	-
Door-to-balloon	k = 19<br>*p* = 0.027	k = 19<br>*p* = 0.904	k = 14<br>*p* = 0.708	k = 12<br>*p* = 0.315	k = 12<br>*p* = 0.973	k = 12<br>*p* = 0.224	-
Time to first medical contact	k = 20<br>*p* = 0.534	k = 20<br>*p* = 0.115	k = 18<br>*p* = 0.422	k = 18<br>*p* = 0.015	k = 13<br>*p* = 0.594	k = 15<br>*p* = 0.677	k = 4<br>*p* = 0.029
Mechanical complications	k = 7<br>*p* = 0.0.589	k = 7<br>*p* = 0.326	k = 6<br>*p* = 0.366	k = 6<br>*p* = 0.186	k = 5<br>*p* = 0.028	k = 5<br>0.967	-
**NSTEMI**	**Age**	**Gender**	**DM**	**Smoking**	**Dyslipidemia**	**Hypertension**	**CKD**
Hospital admissions<br>OR	k = 5<br>*p* = 0.0551	k = 5<br>*p* = 0.784	-	-	-	-	-
